# Supplementary figures and images for: Bacterial cell widening alters periplasmic size and activates envelope stress responses
Source: EMBO J. 2025 Sep 3;44(20):5816–33. doi: 10.1038/s44318-025-00534-w (PMC12528386; doi:10.1038/s44318-025-00534-w)

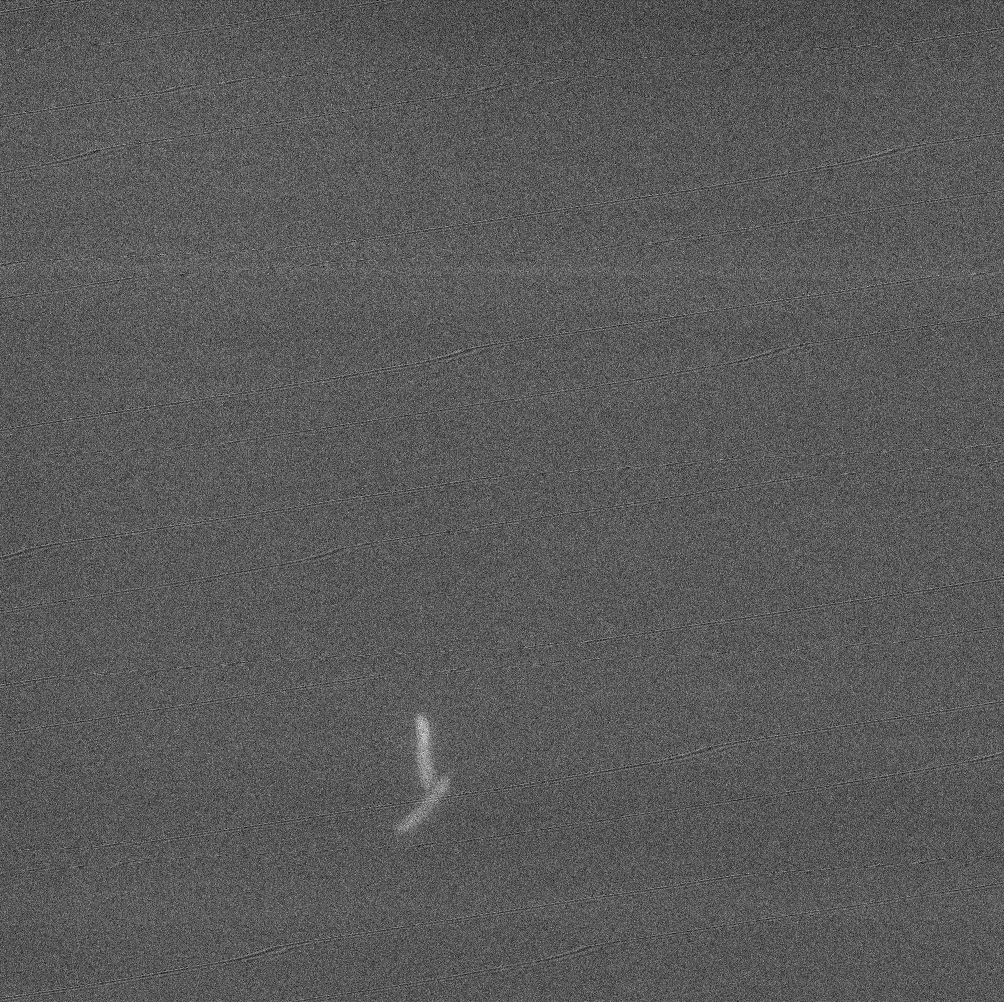

Supplement: Supplementary file 2 — Source data Fig. 1 [file 44318_2025_534_MOESM2_ESM.zip › Figure 1/1C/0ugmL/img_000000000_ETGFP_000.tif]

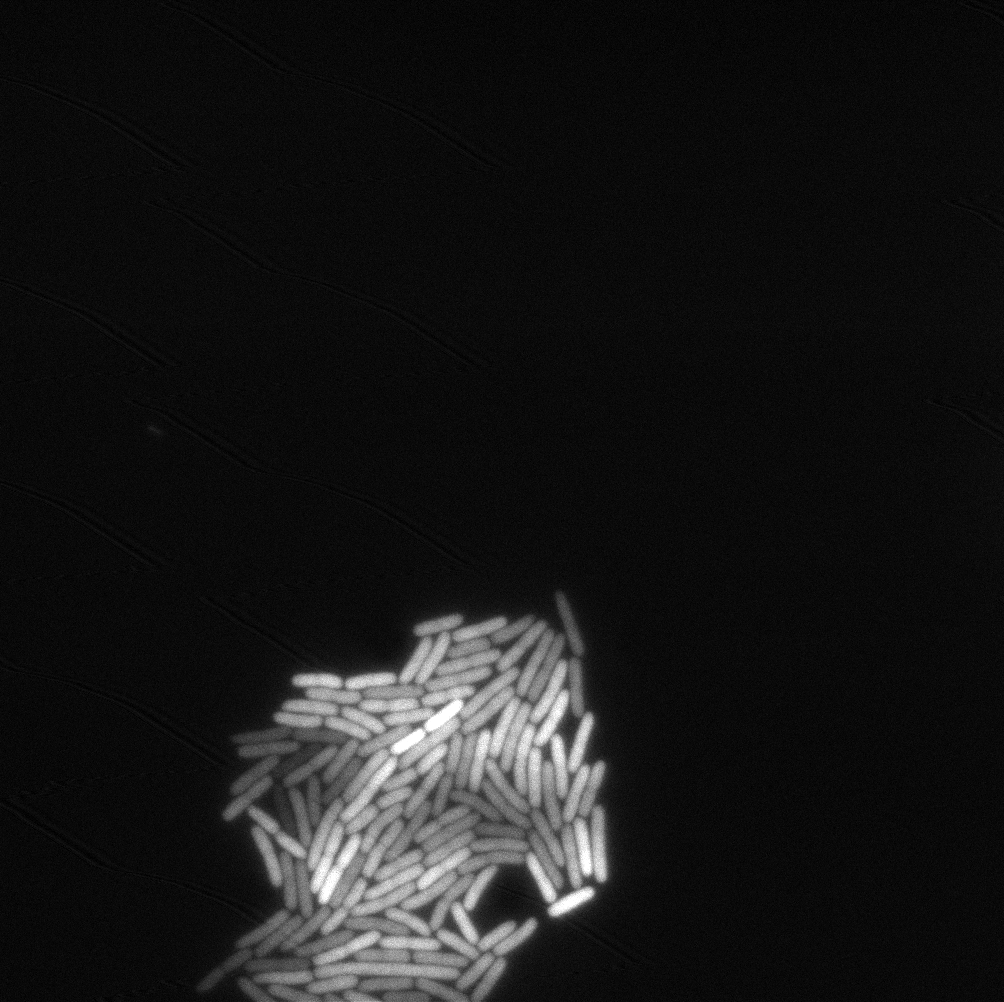

Supplement: Supplementary file 2 — Source data Fig. 1 [file 44318_2025_534_MOESM2_ESM.zip › Figure 1/1C/0ugmL/img_000000060_ETGFP_000.tif]

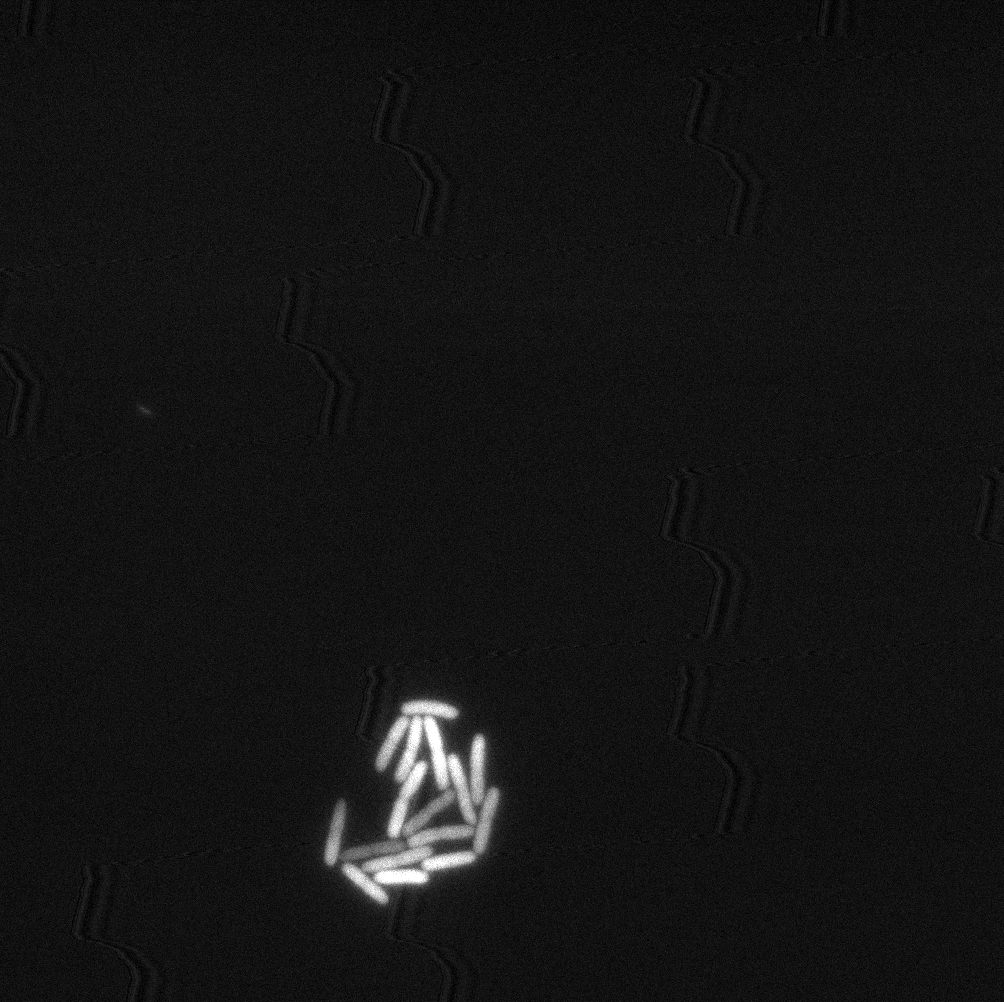

Supplement: Supplementary file 2 — Source data Fig. 1 [file 44318_2025_534_MOESM2_ESM.zip › Figure 1/1C/0ugmL/img_000000030_ETGFP_000.tif]

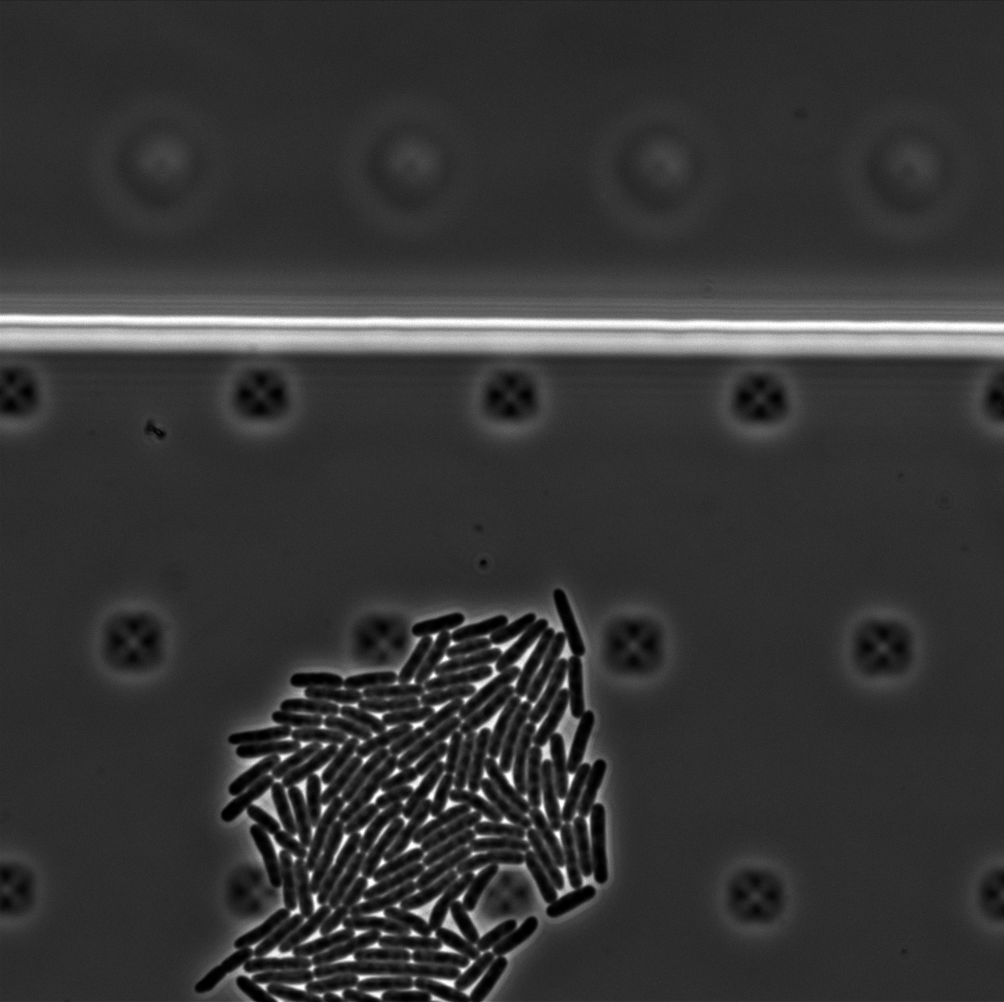

Supplement: Supplementary file 2 — Source data Fig. 1 [file 44318_2025_534_MOESM2_ESM.zip › Figure 1/1C/0ugmL/img_000000060_Phase (ETGFP)_000.tif]

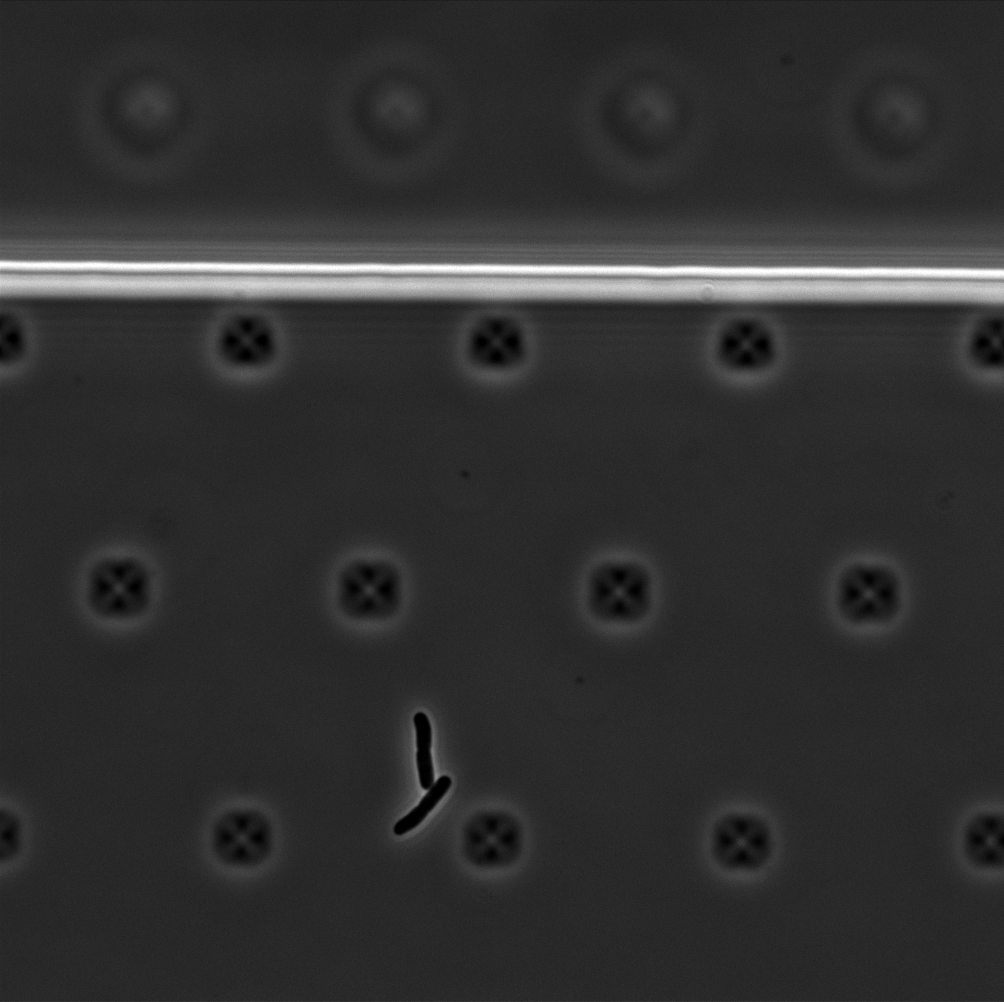

Supplement: Supplementary file 2 — Source data Fig. 1 [file 44318_2025_534_MOESM2_ESM.zip › Figure 1/1C/0ugmL/img_000000000_Phase (ETGFP)_000.tif]

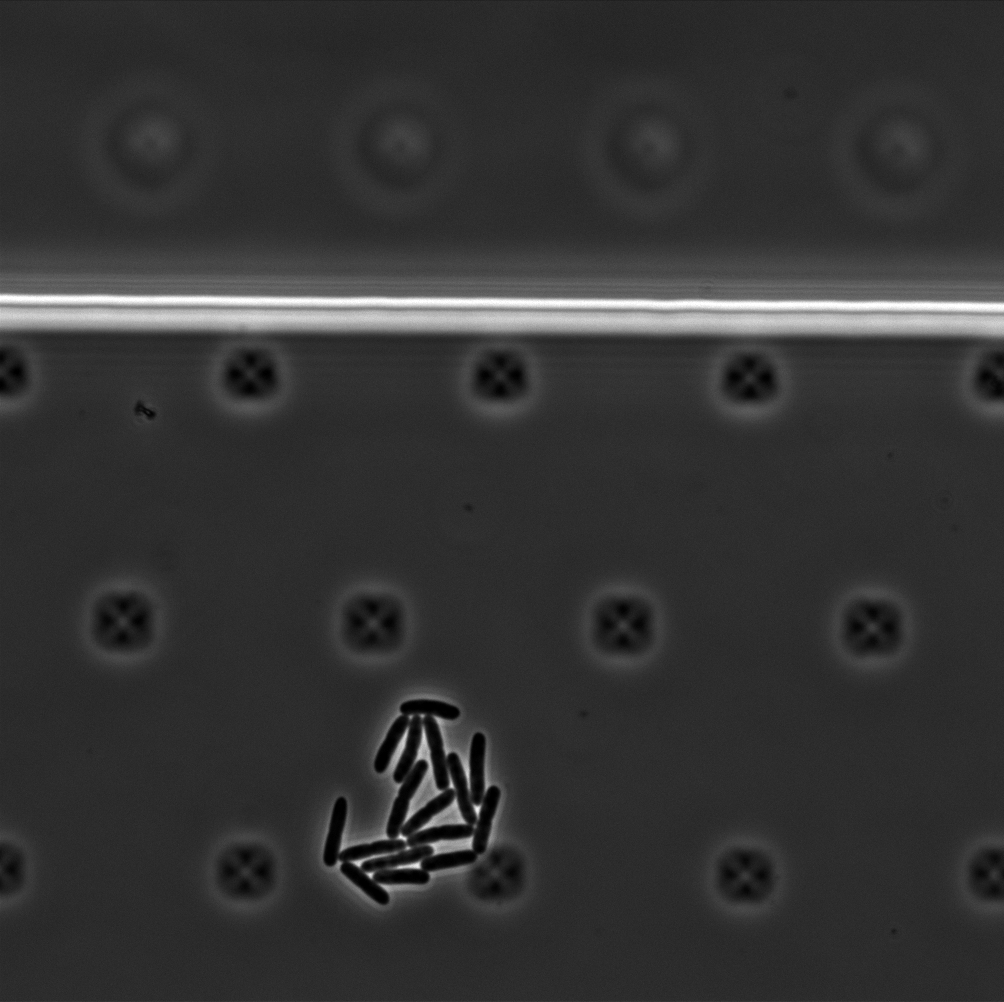

Supplement: Supplementary file 2 — Source data Fig. 1 [file 44318_2025_534_MOESM2_ESM.zip › Figure 1/1C/0ugmL/img_000000030_Phase (ETGFP)_000.tif]

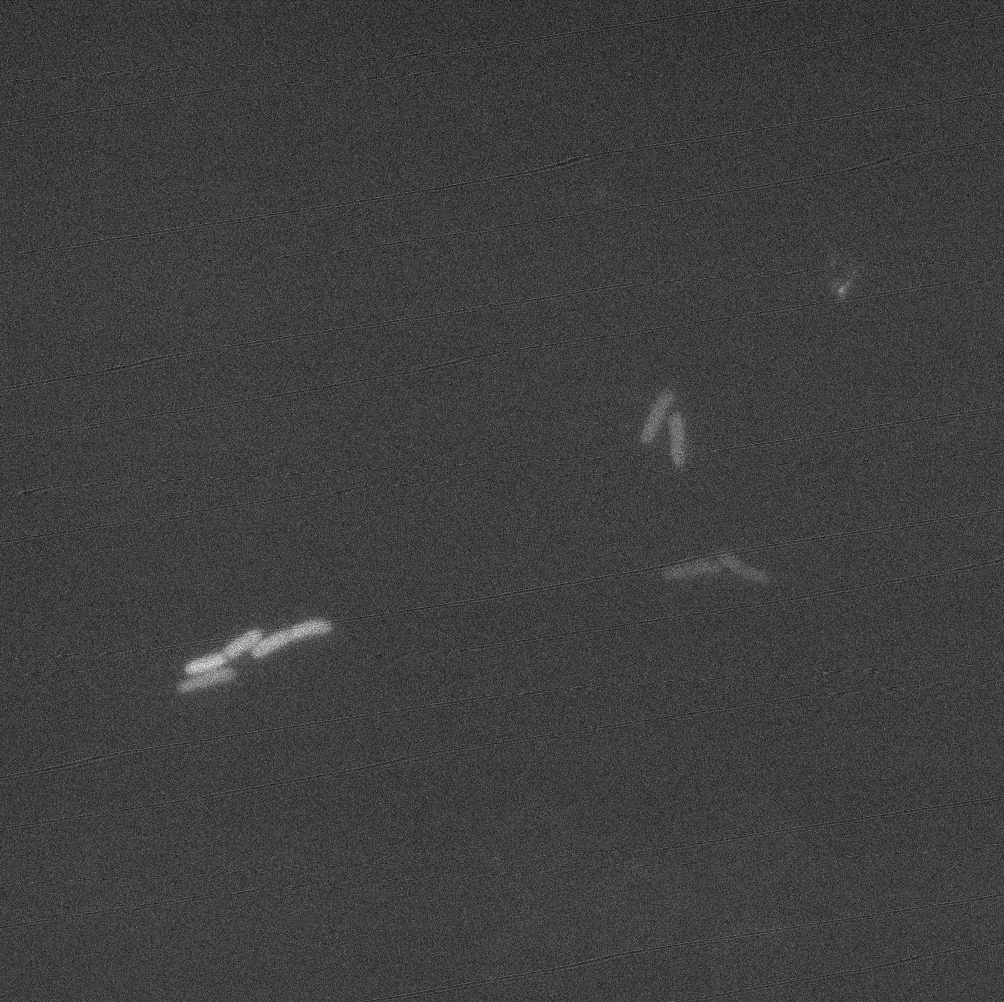

Supplement: Supplementary file 2 — Source data Fig. 1 [file 44318_2025_534_MOESM2_ESM.zip › Figure 1/1C/2ugmL/img_000000000_ETGFP_000.tif]

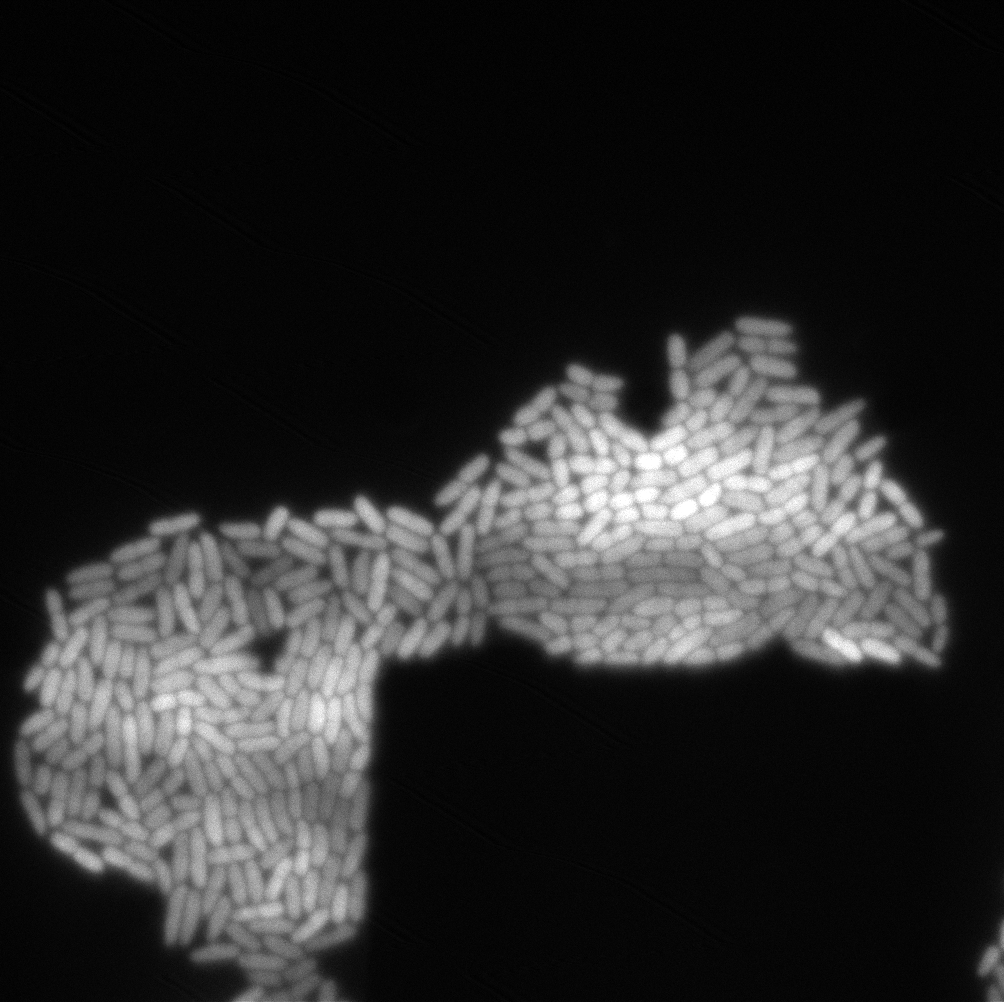

Supplement: Supplementary file 2 — Source data Fig. 1 [file 44318_2025_534_MOESM2_ESM.zip › Figure 1/1C/2ugmL/img_000000060_ETGFP_000.tif]

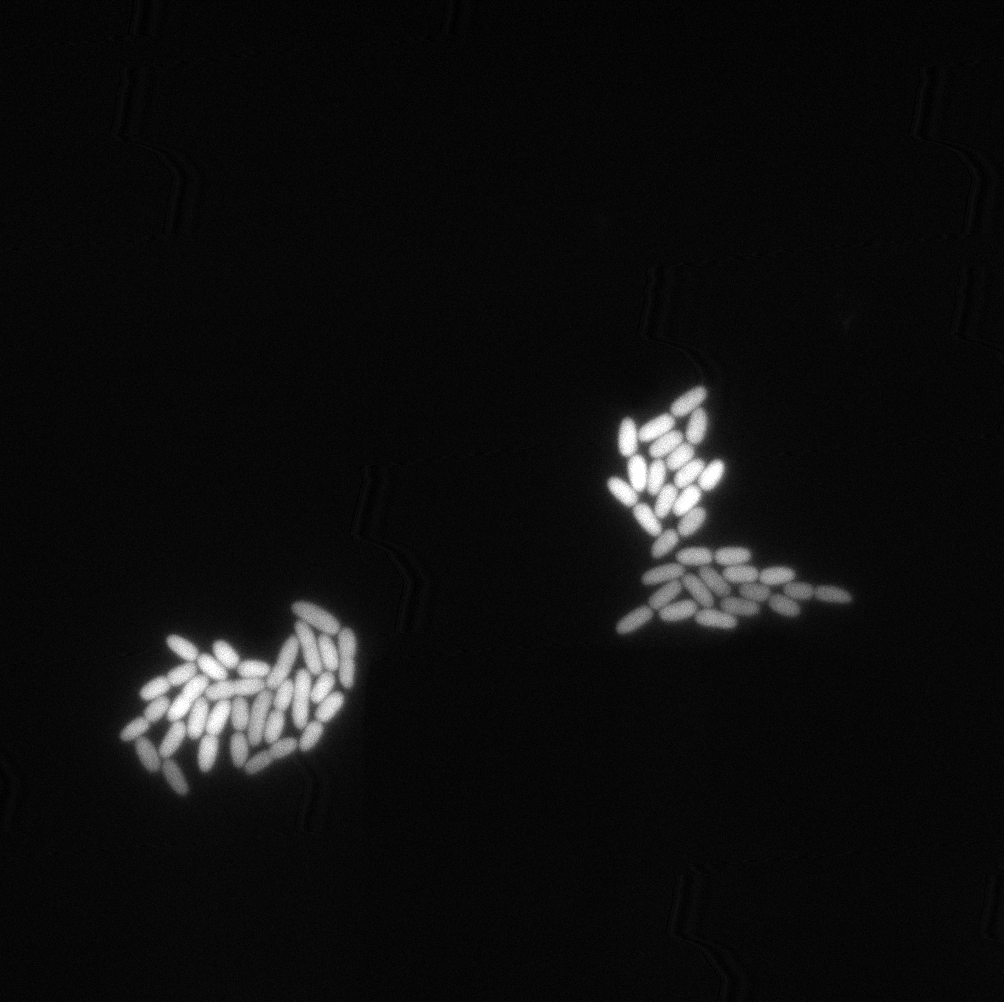

Supplement: Supplementary file 2 — Source data Fig. 1 [file 44318_2025_534_MOESM2_ESM.zip › Figure 1/1C/2ugmL/img_000000030_ETGFP_000.tif]

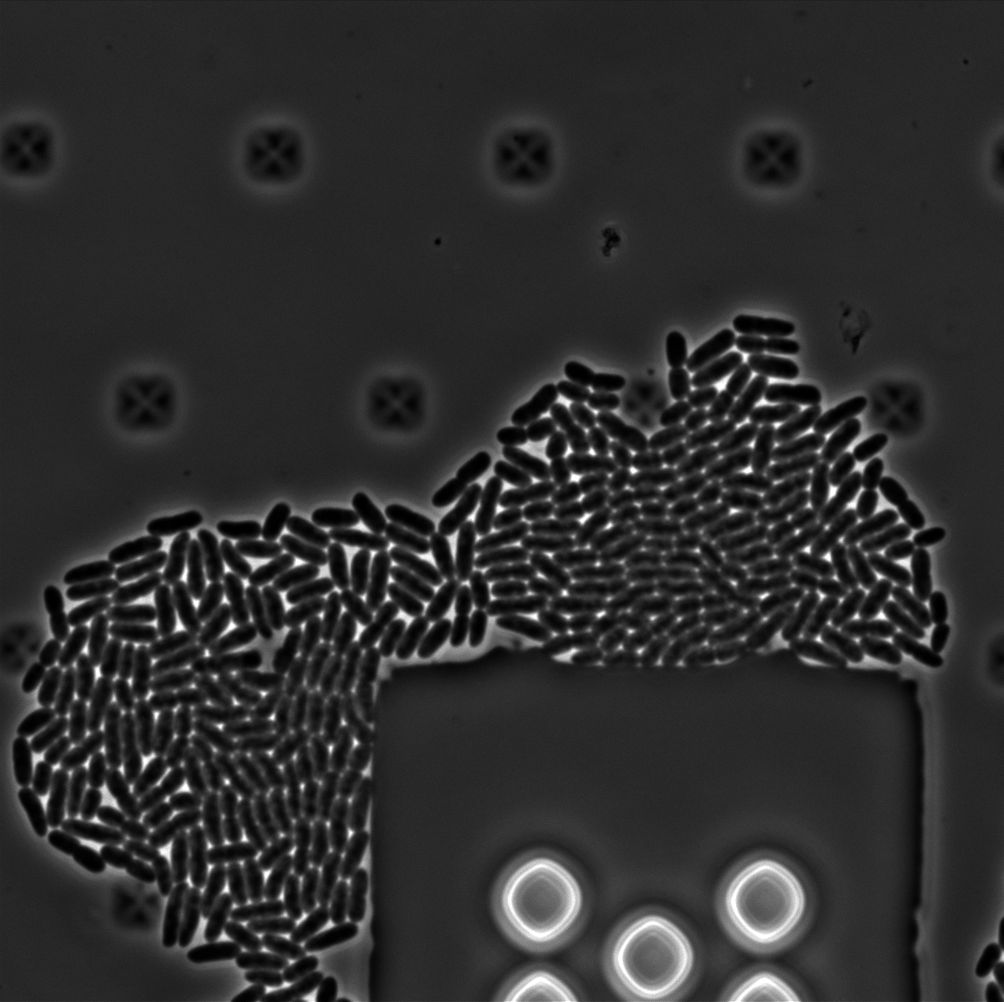

Supplement: Supplementary file 2 — Source data Fig. 1 [file 44318_2025_534_MOESM2_ESM.zip › Figure 1/1C/2ugmL/img_000000060_Phase (ETGFP)_000.tif]

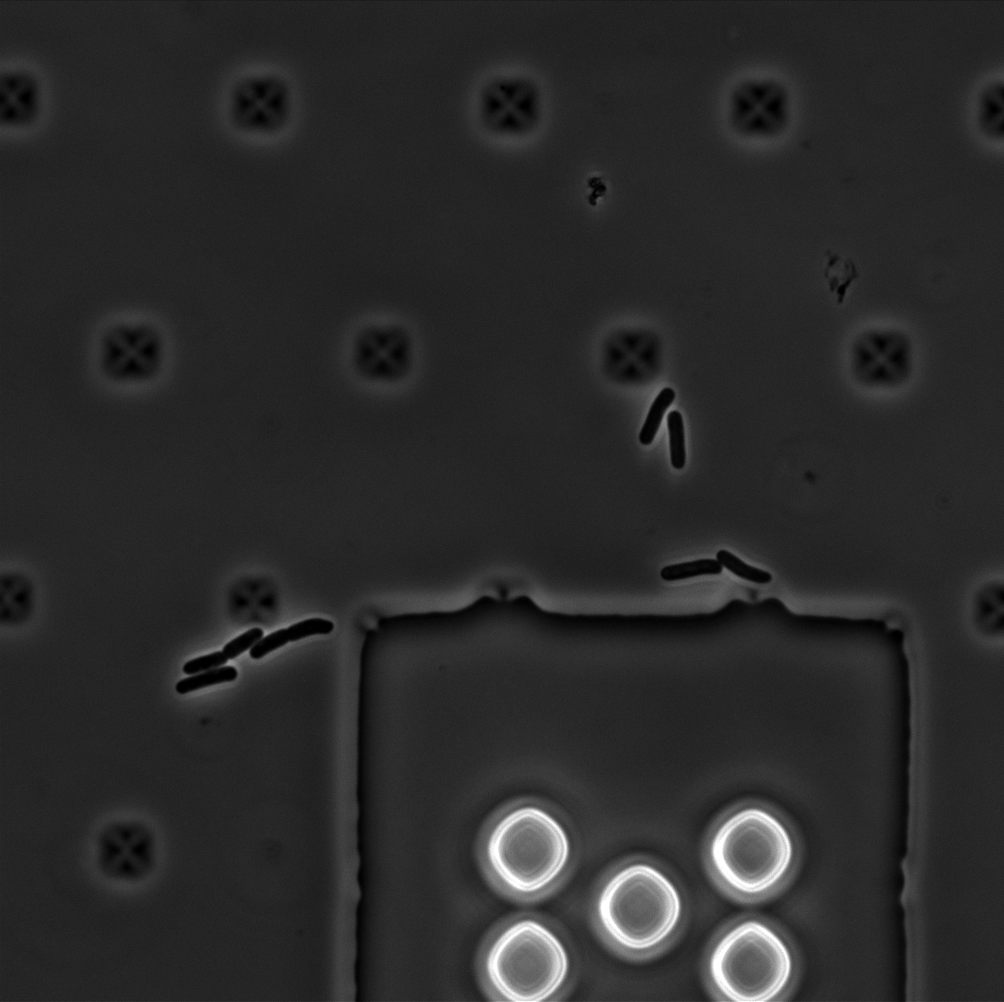

Supplement: Supplementary file 2 — Source data Fig. 1 [file 44318_2025_534_MOESM2_ESM.zip › Figure 1/1C/2ugmL/img_000000000_Phase (ETGFP)_000.tif]

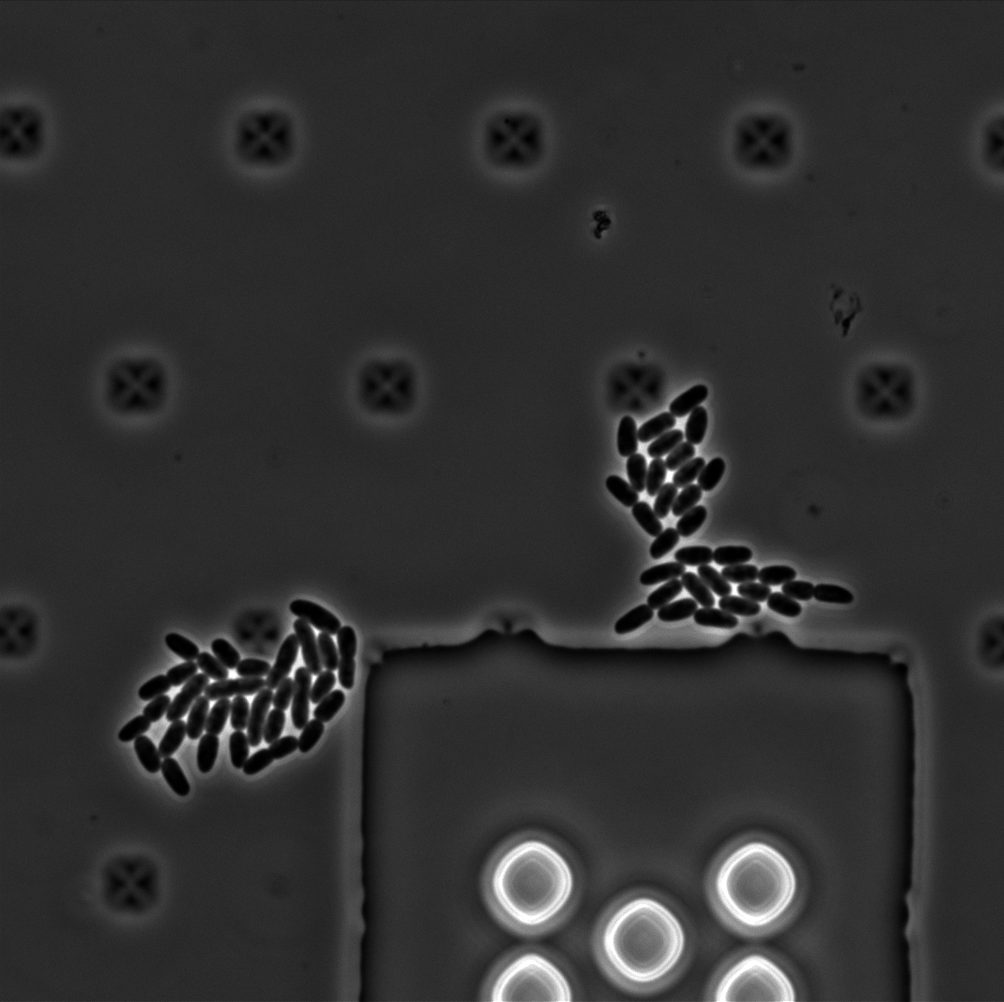

Supplement: Supplementary file 2 — Source data Fig. 1 [file 44318_2025_534_MOESM2_ESM.zip › Figure 1/1C/2ugmL/img_000000030_Phase (ETGFP)_000.tif]

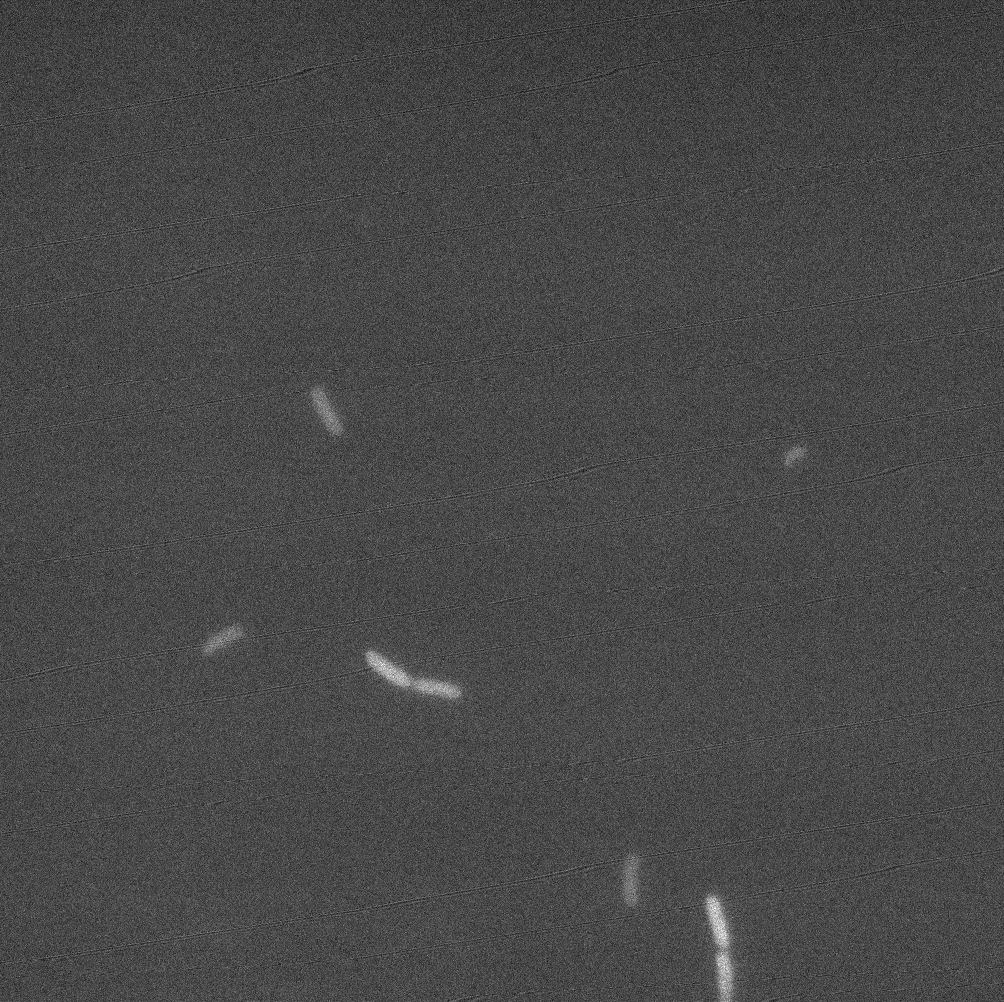

Supplement: Supplementary file 2 — Source data Fig. 1 [file 44318_2025_534_MOESM2_ESM.zip › Figure 1/1C/5ugmL/img_000000000_ETGFP_000.tif]

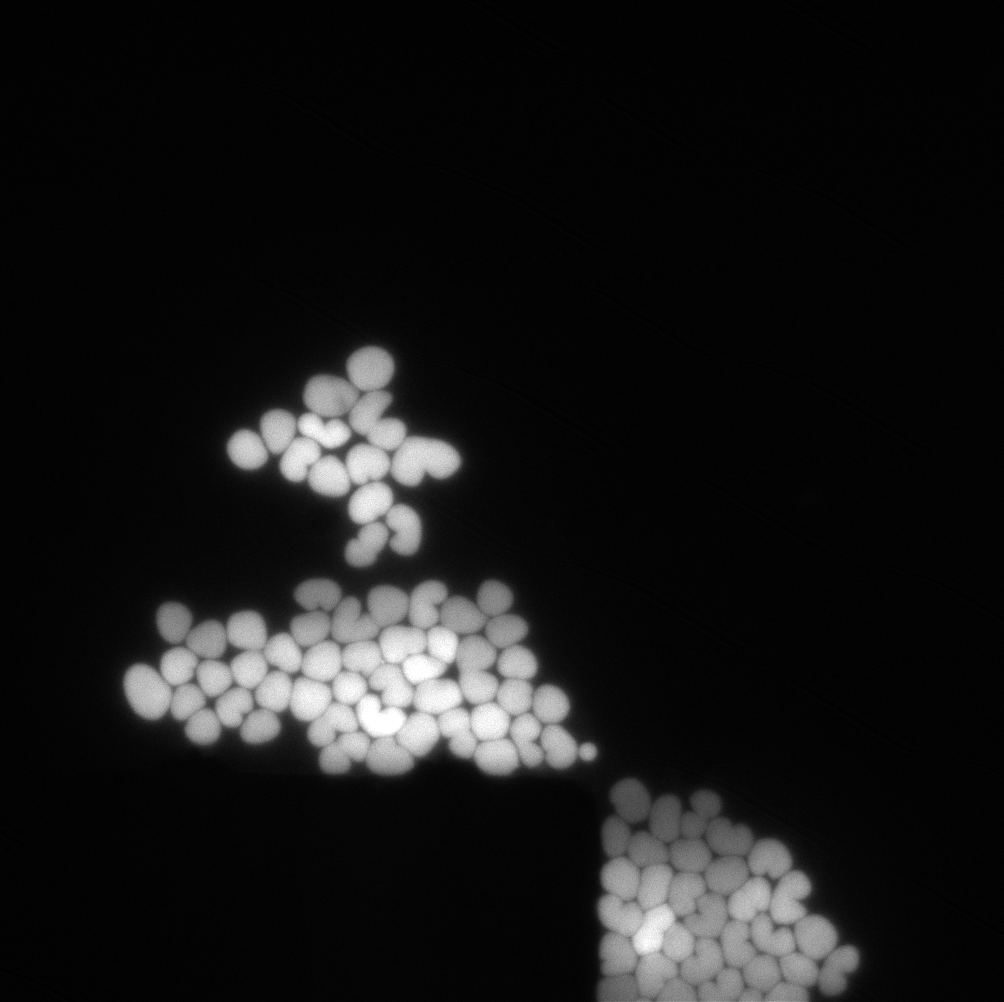

Supplement: Supplementary file 2 — Source data Fig. 1 [file 44318_2025_534_MOESM2_ESM.zip › Figure 1/1C/5ugmL/img_000000060_ETGFP_000.tif]

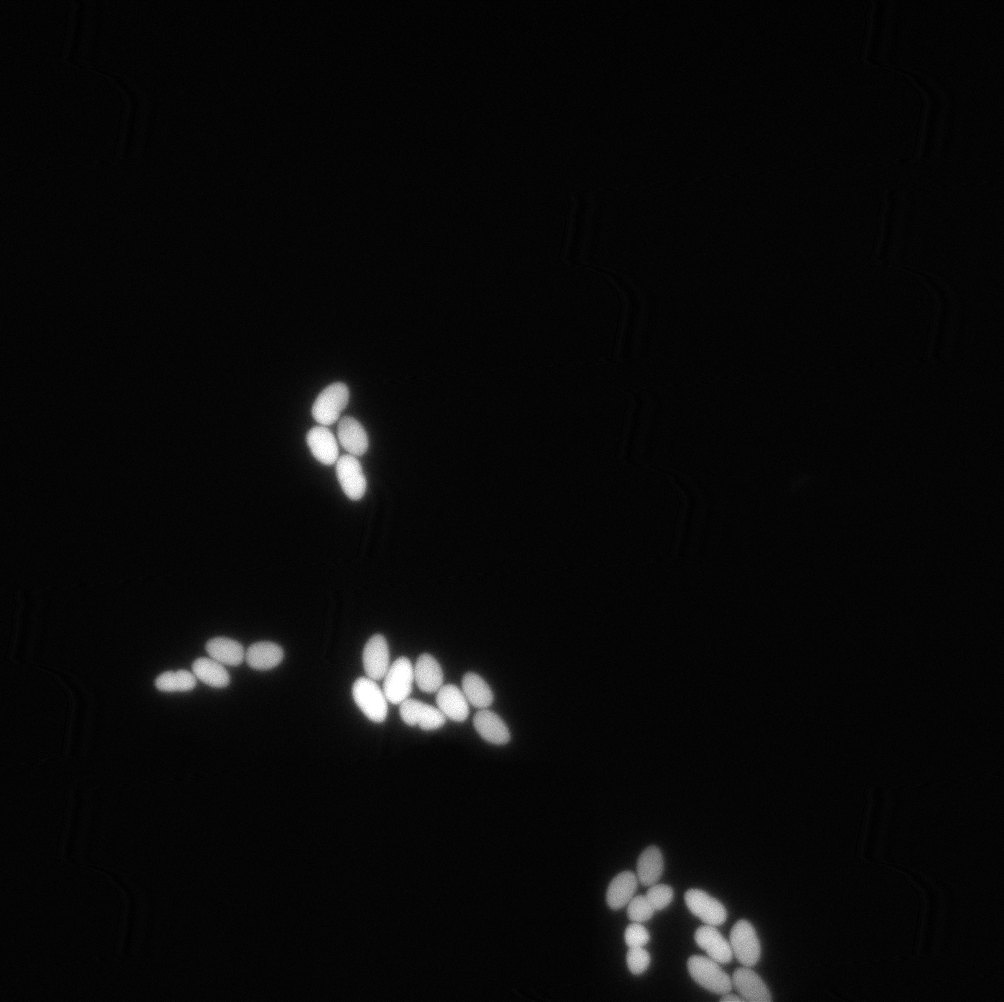

Supplement: Supplementary file 2 — Source data Fig. 1 [file 44318_2025_534_MOESM2_ESM.zip › Figure 1/1C/5ugmL/img_000000030_ETGFP_000.tif]

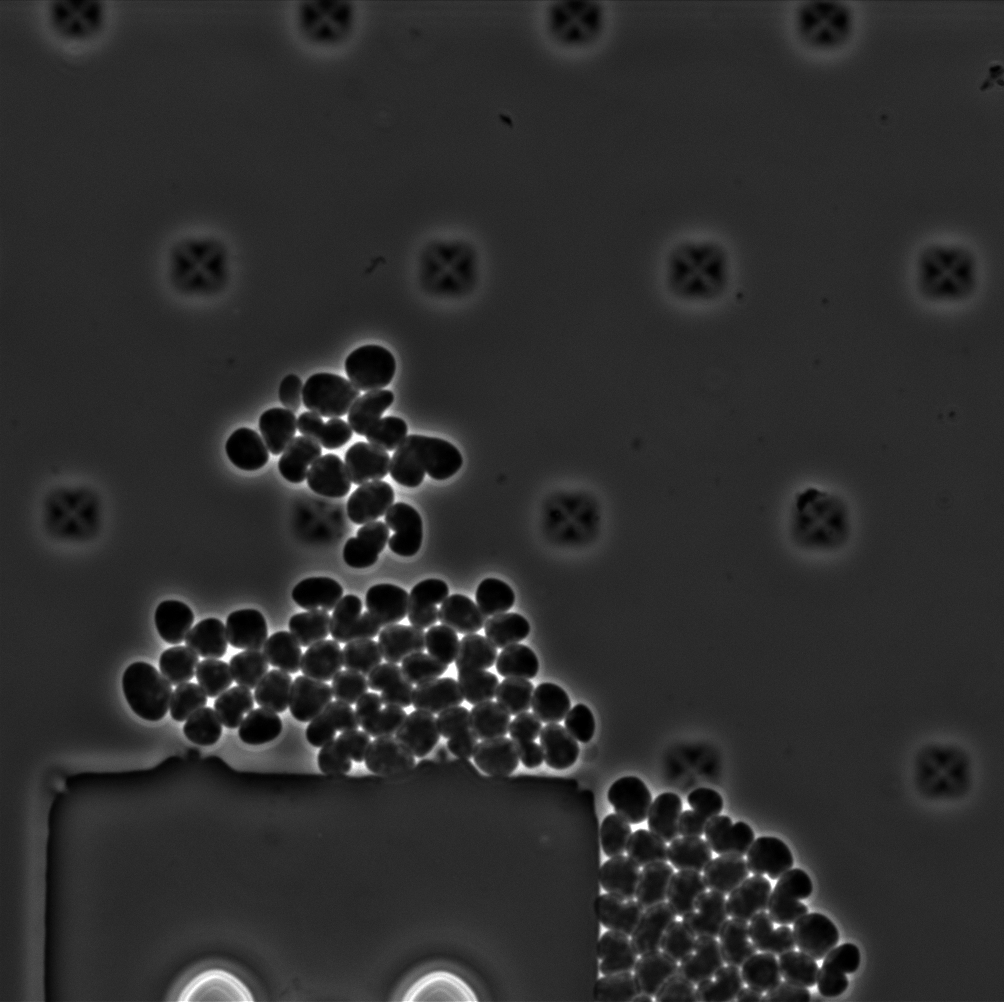

Supplement: Supplementary file 2 — Source data Fig. 1 [file 44318_2025_534_MOESM2_ESM.zip › Figure 1/1C/5ugmL/img_000000060_Phase (ETGFP)_000.tif]

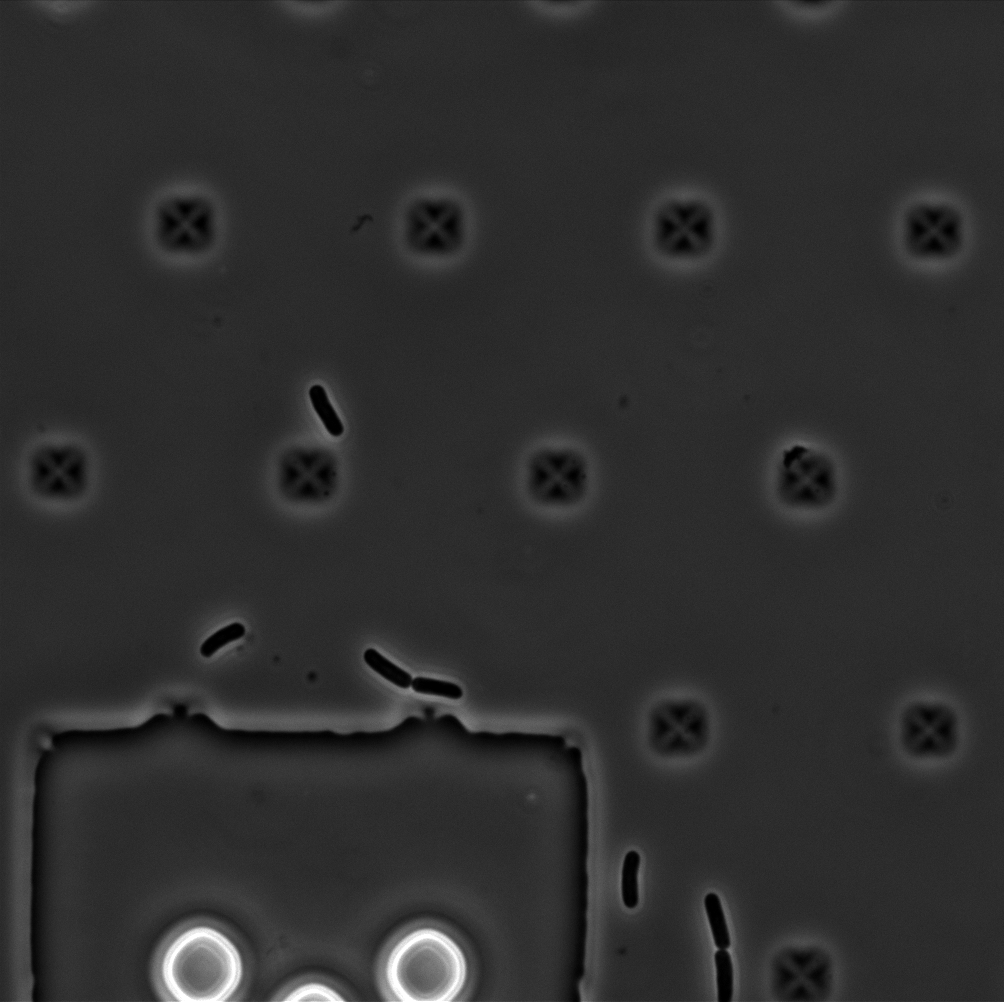

Supplement: Supplementary file 2 — Source data Fig. 1 [file 44318_2025_534_MOESM2_ESM.zip › Figure 1/1C/5ugmL/img_000000000_Phase (ETGFP)_000.tif]

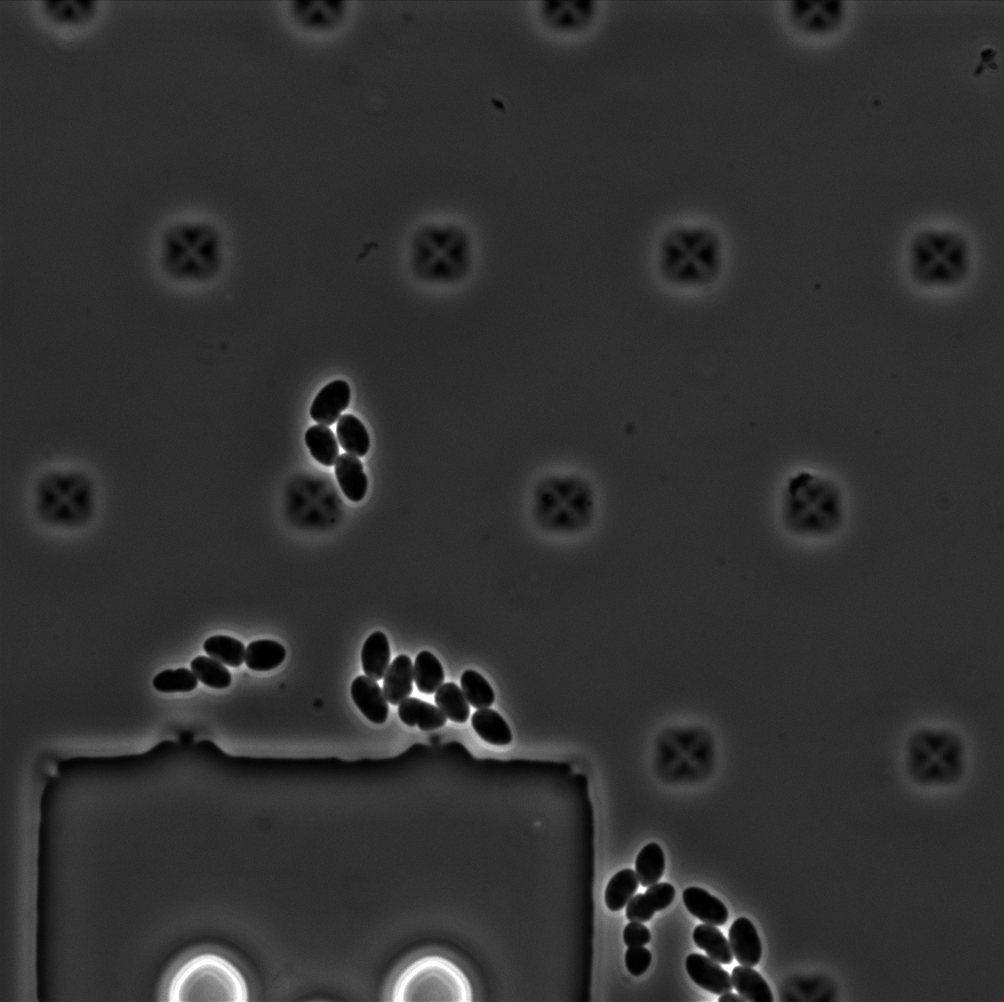

Supplement: Supplementary file 2 — Source data Fig. 1 [file 44318_2025_534_MOESM2_ESM.zip › Figure 1/1C/5ugmL/img_000000030_Phase (ETGFP)_000.tif]

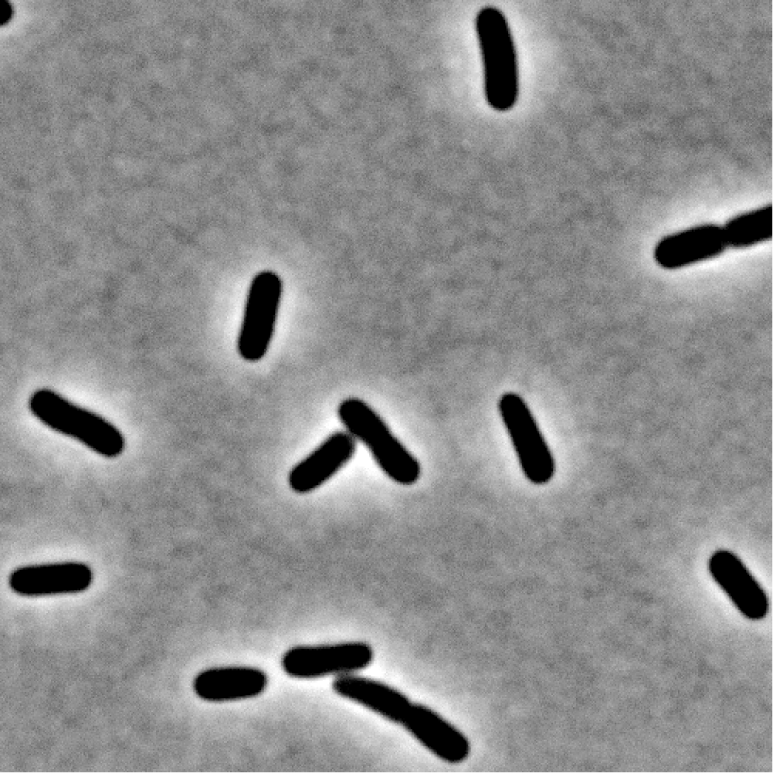

Supplement: Supplementary file 3 — Source data Fig. 2 [file 44318_2025_534_MOESM3_ESM.zip › Figure 2/2C/opgH.tif]

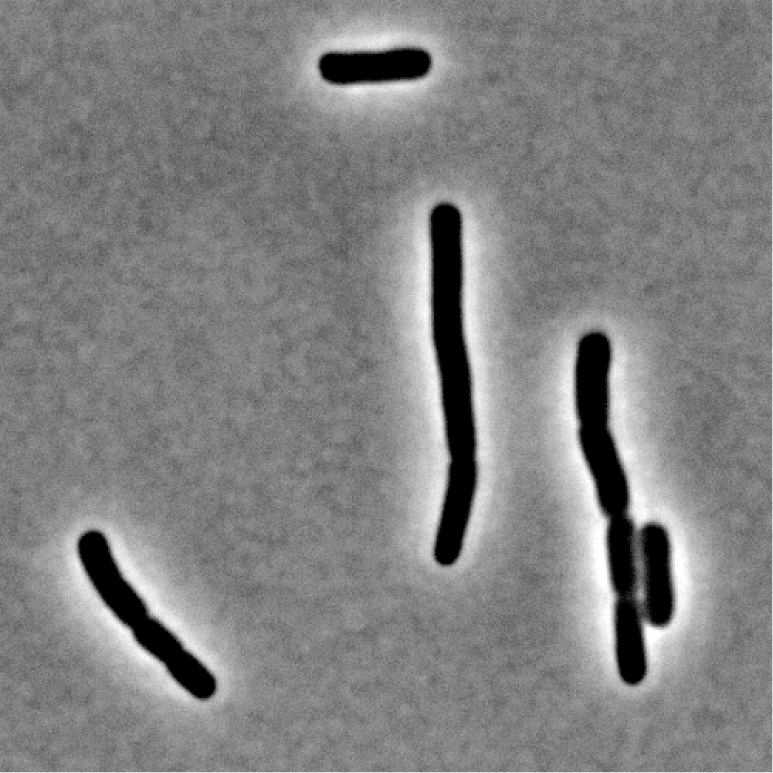

Supplement: Supplementary file 3 — Source data Fig. 2 [file 44318_2025_534_MOESM3_ESM.zip › Figure 2/2C/tolB.tif]

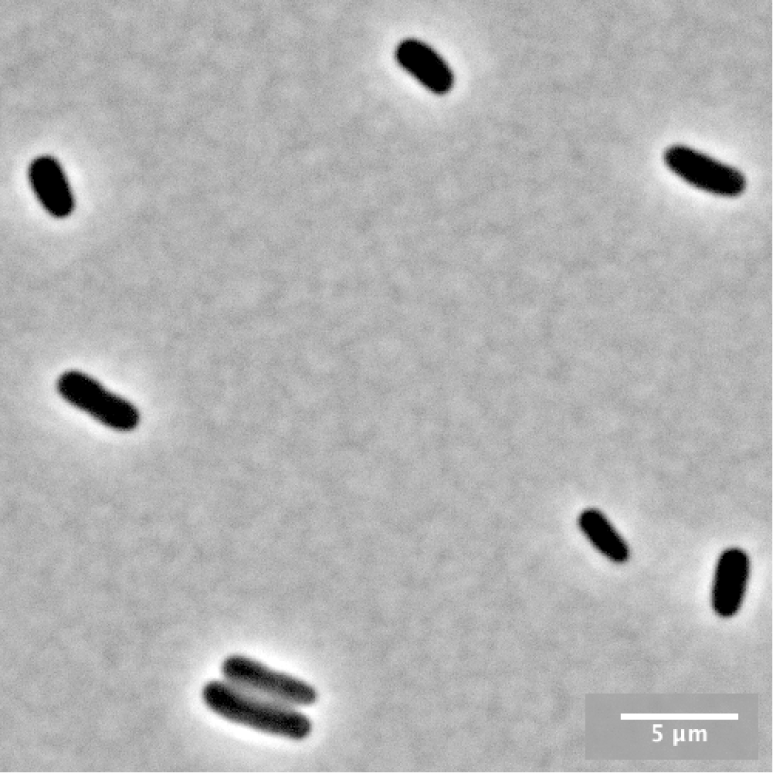

Supplement: Supplementary file 3 — Source data Fig. 2 [file 44318_2025_534_MOESM3_ESM.zip › Figure 2/2C/pgm.tif]

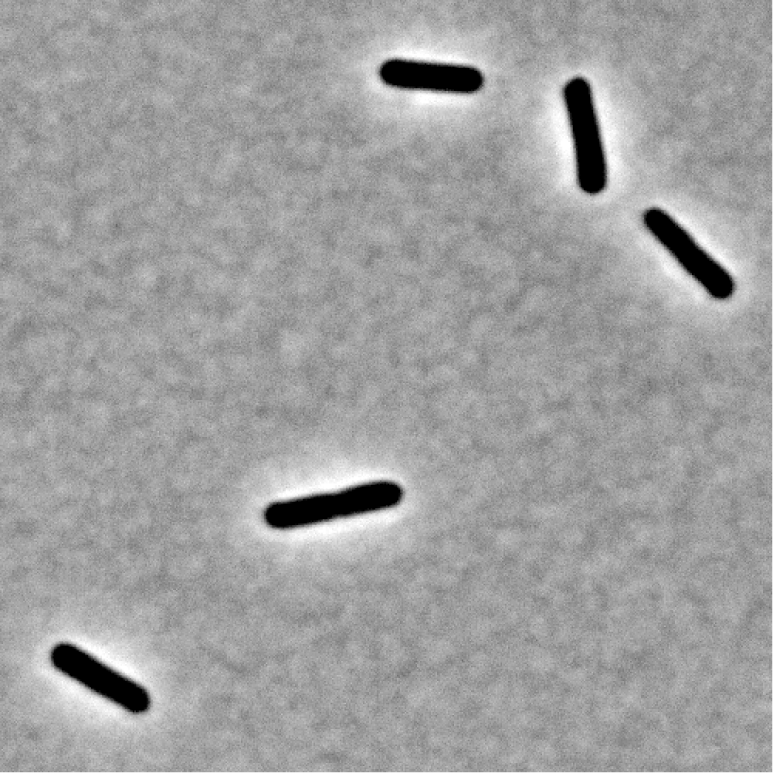

Supplement: Supplementary file 3 — Source data Fig. 2 [file 44318_2025_534_MOESM3_ESM.zip › Figure 2/2C/WT.tif]

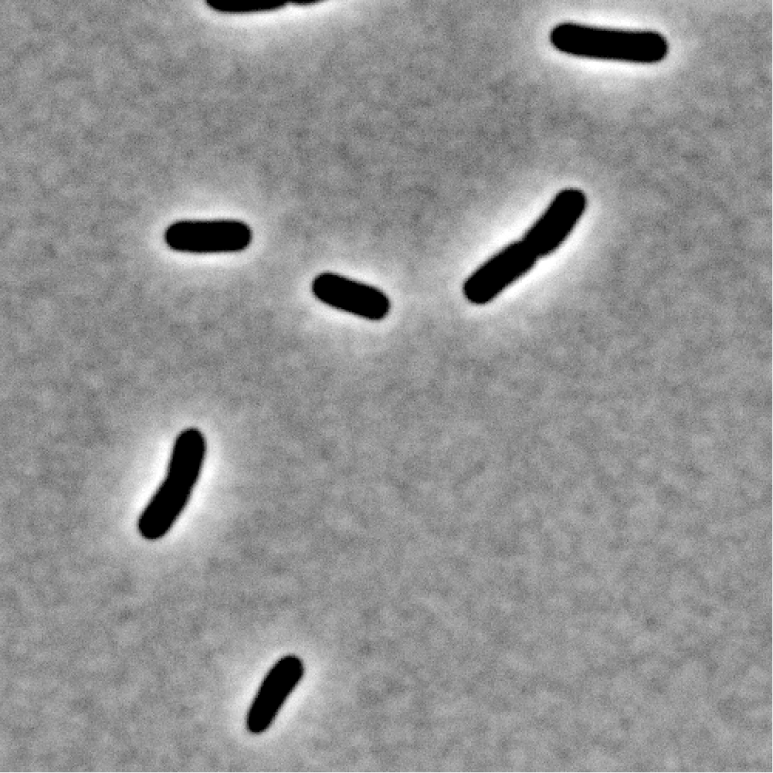

Supplement: Supplementary file 3 — Source data Fig. 2 [file 44318_2025_534_MOESM3_ESM.zip › Figure 2/2C/opgG.tif]

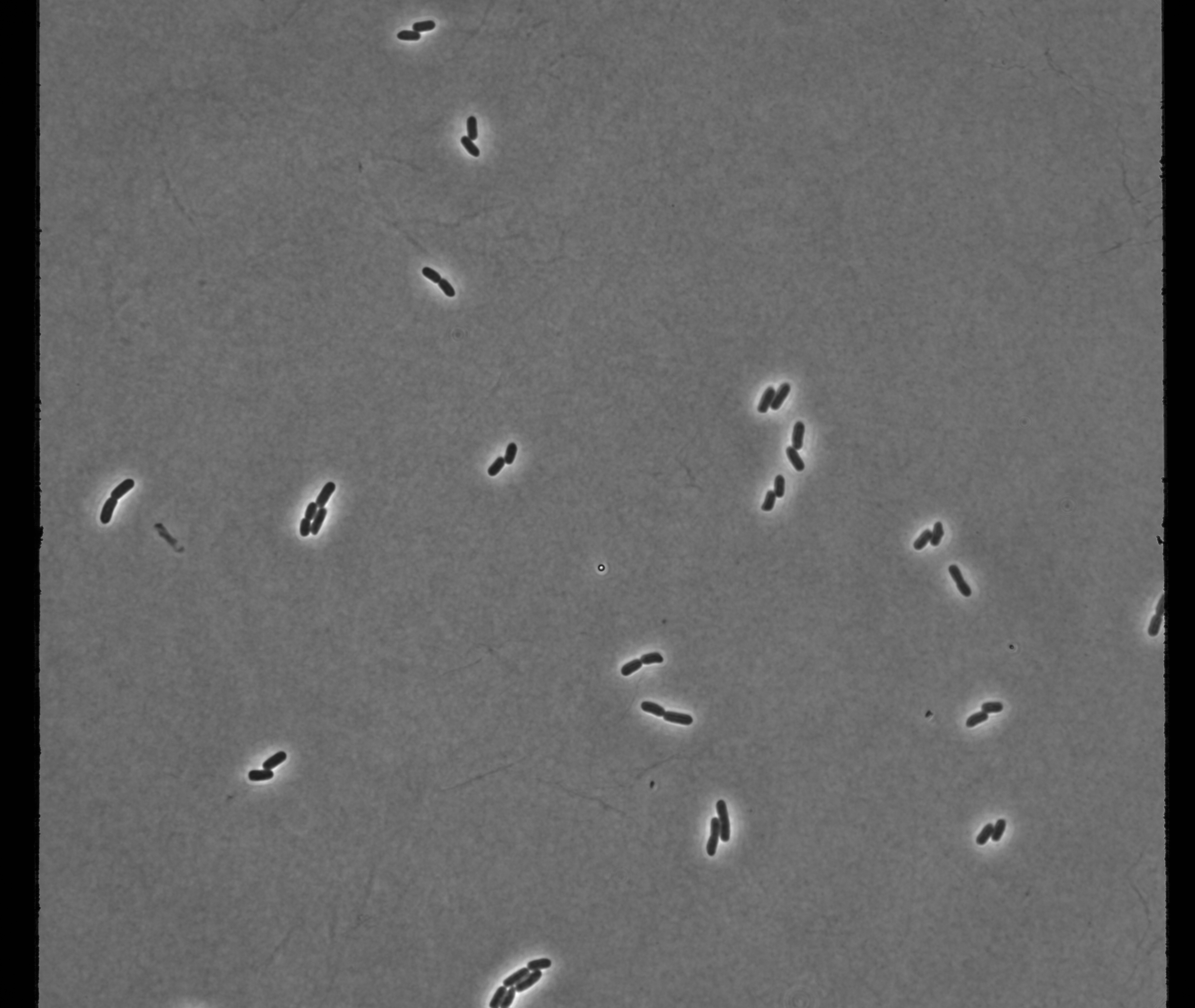

Supplement: Supplementary file 3 — Source data Fig. 2 [file 44318_2025_534_MOESM3_ESM.zip › Figure 2/2A/V236A/img_000000000_Phase_000.tif]

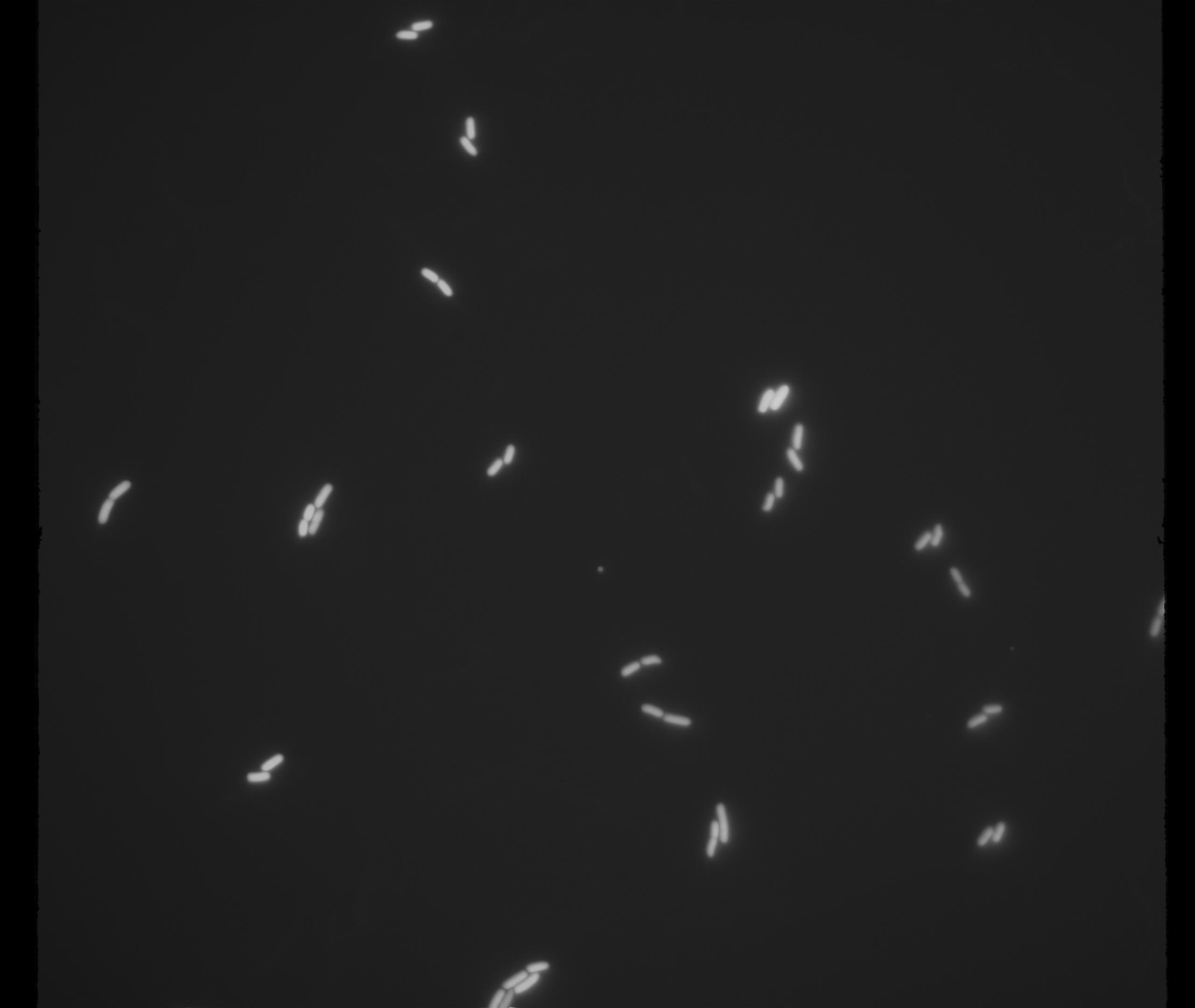

Supplement: Supplementary file 3 — Source data Fig. 2 [file 44318_2025_534_MOESM3_ESM.zip › Figure 2/2A/V236A/img_000000000_EGFP_000.tif]

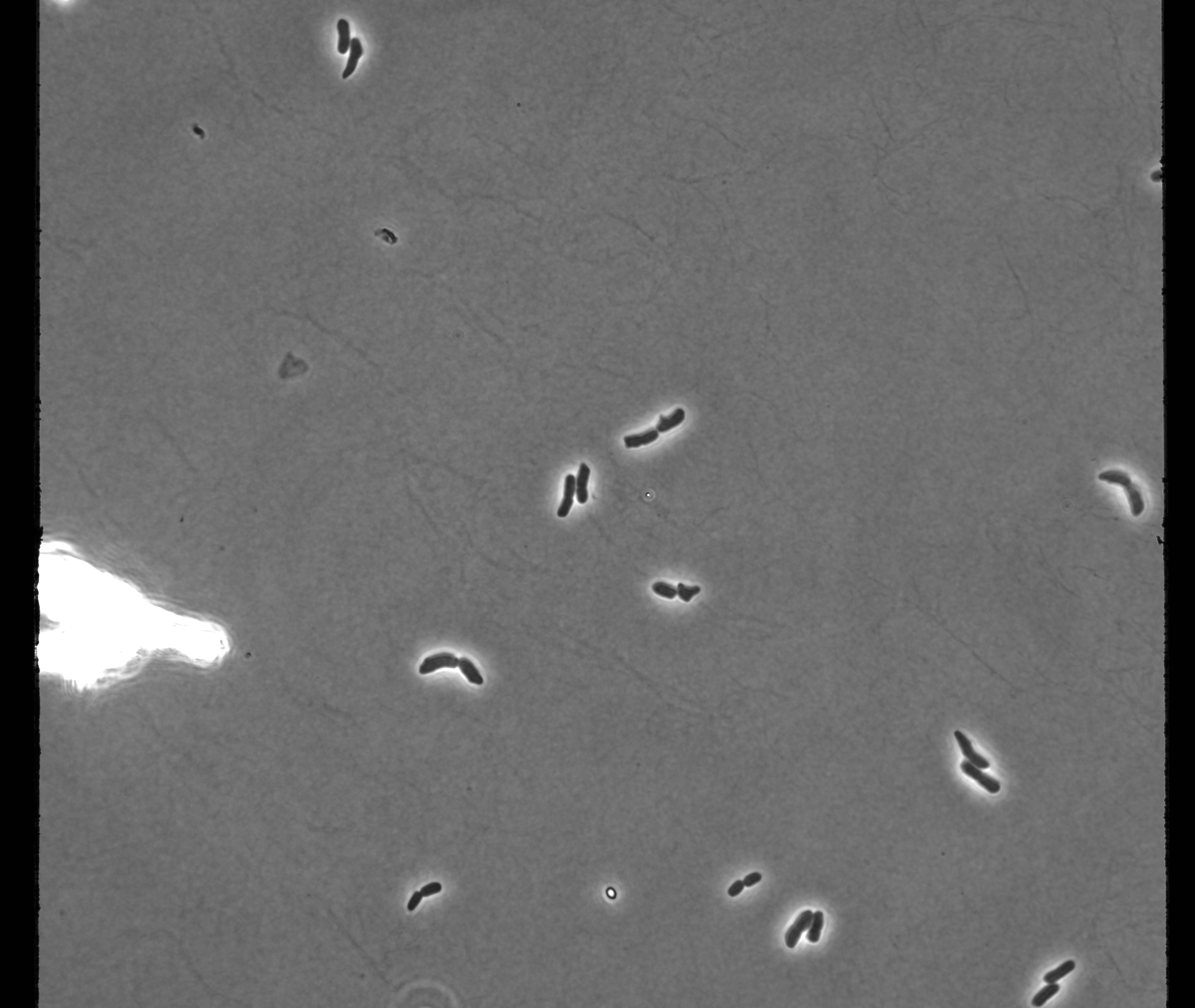

Supplement: Supplementary file 3 — Source data Fig. 2 [file 44318_2025_534_MOESM3_ESM.zip › Figure 2/2A/S10P/img_000000000_Phase_000.tif]

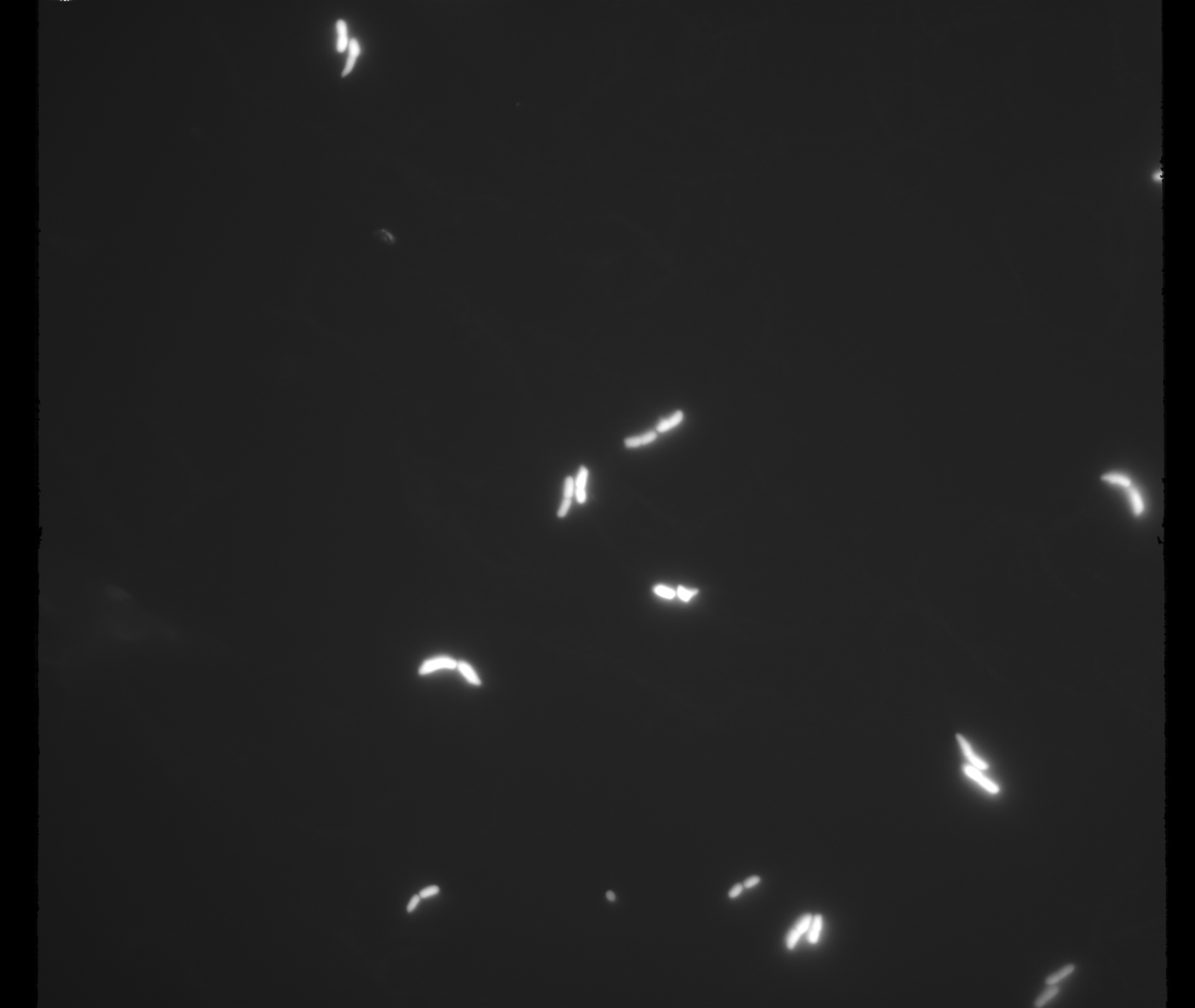

Supplement: Supplementary file 3 — Source data Fig. 2 [file 44318_2025_534_MOESM3_ESM.zip › Figure 2/2A/S10P/img_000000000_EGFP_000.tif]

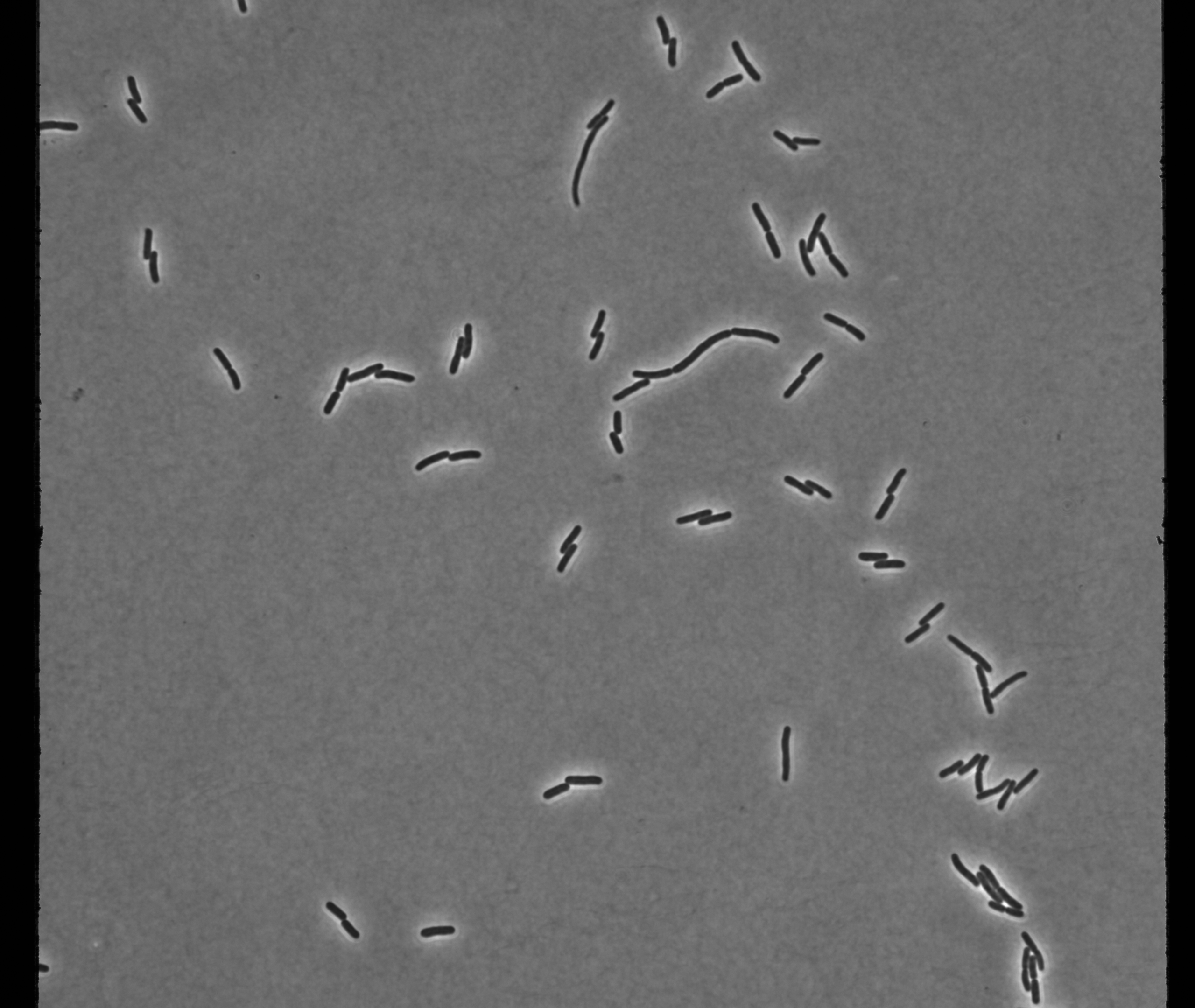

Supplement: Supplementary file 3 — Source data Fig. 2 [file 44318_2025_534_MOESM3_ESM.zip › Figure 2/2A/D78V/img_000000000_Phase_000.tif]

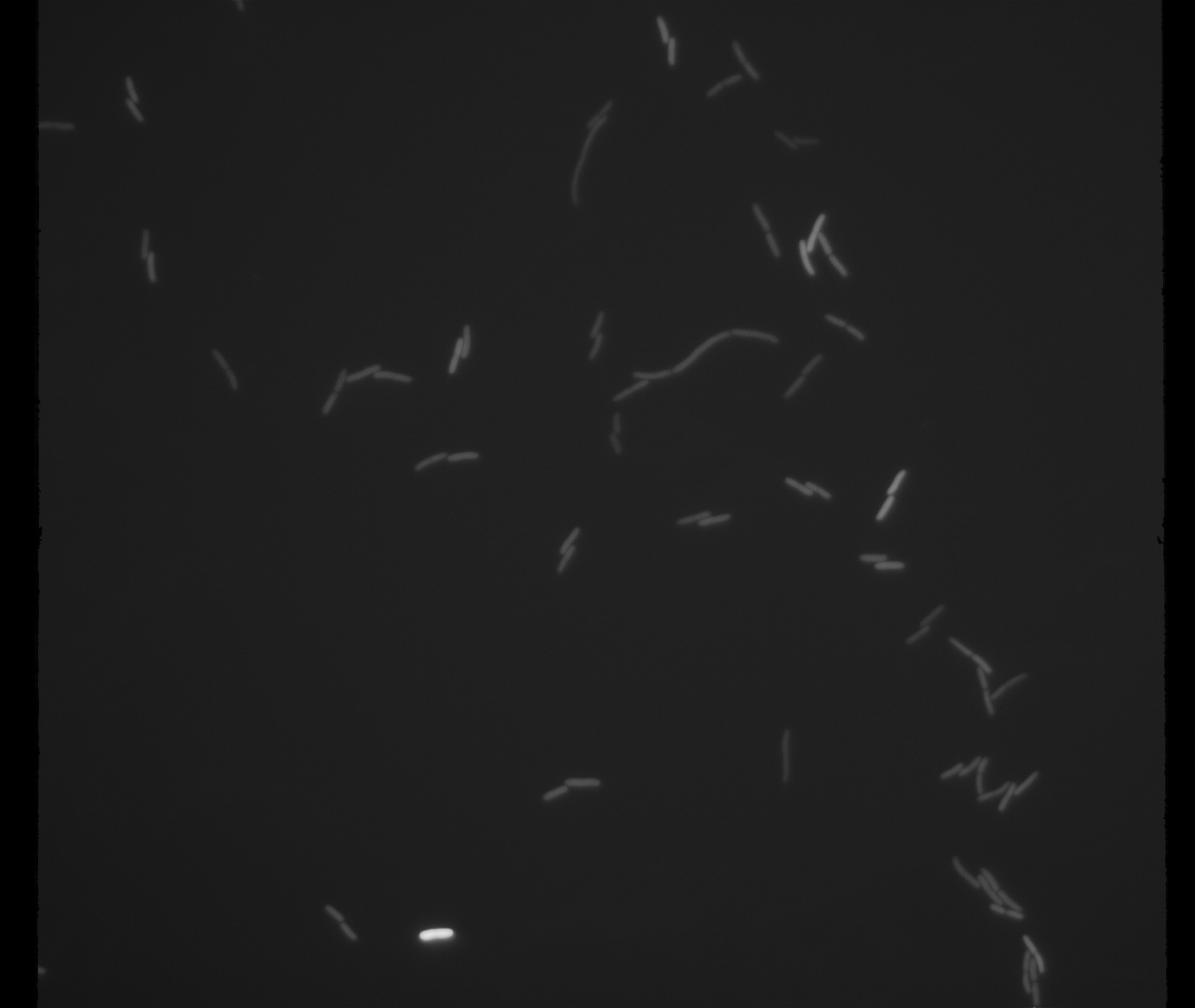

Supplement: Supplementary file 3 — Source data Fig. 2 [file 44318_2025_534_MOESM3_ESM.zip › Figure 2/2A/D78V/img_000000000_EGFP_000.tif]

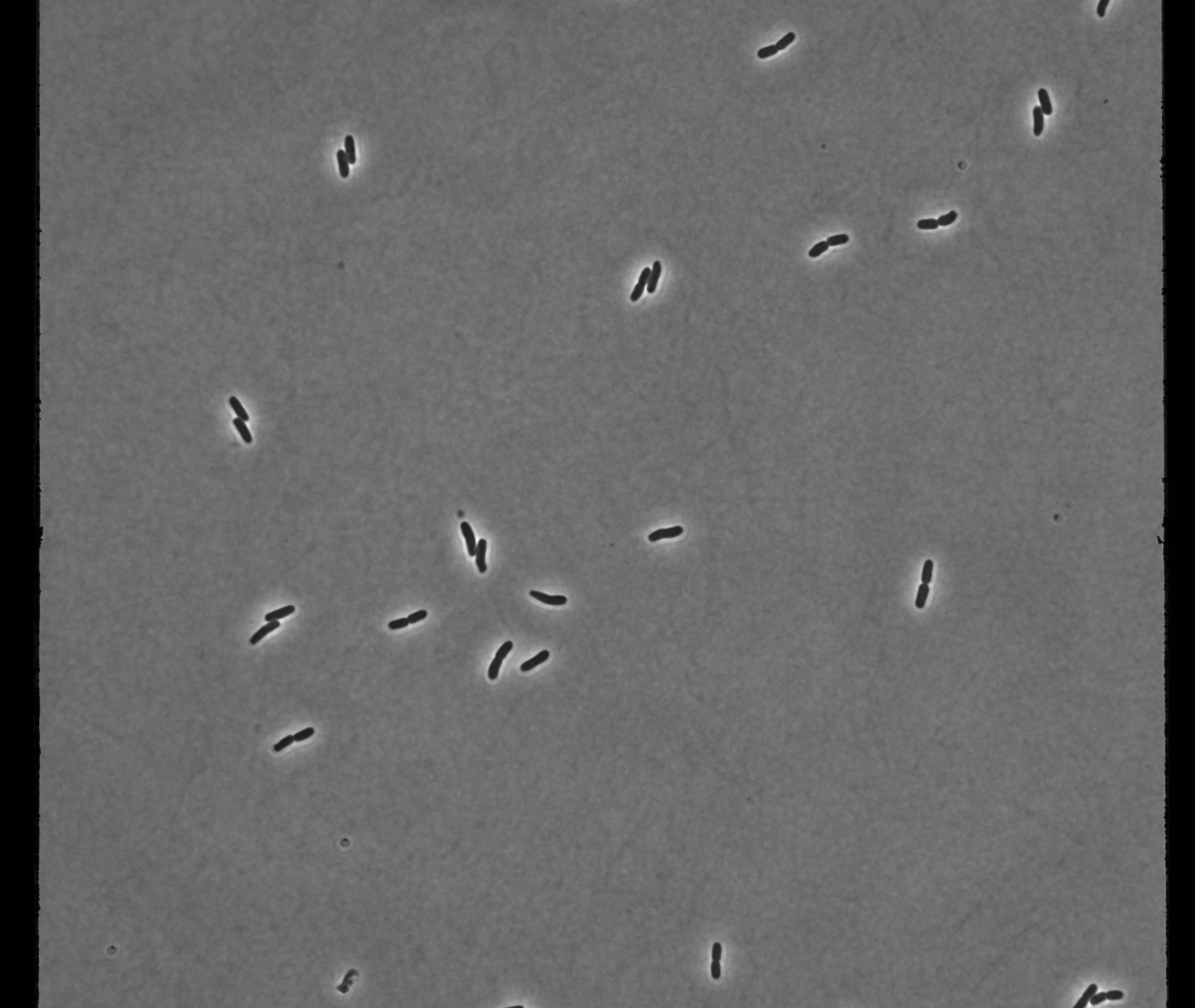

Supplement: Supplementary file 3 — Source data Fig. 2 [file 44318_2025_534_MOESM3_ESM.zip › Figure 2/2A/V316A/img_000000000_Phase_000.tif]

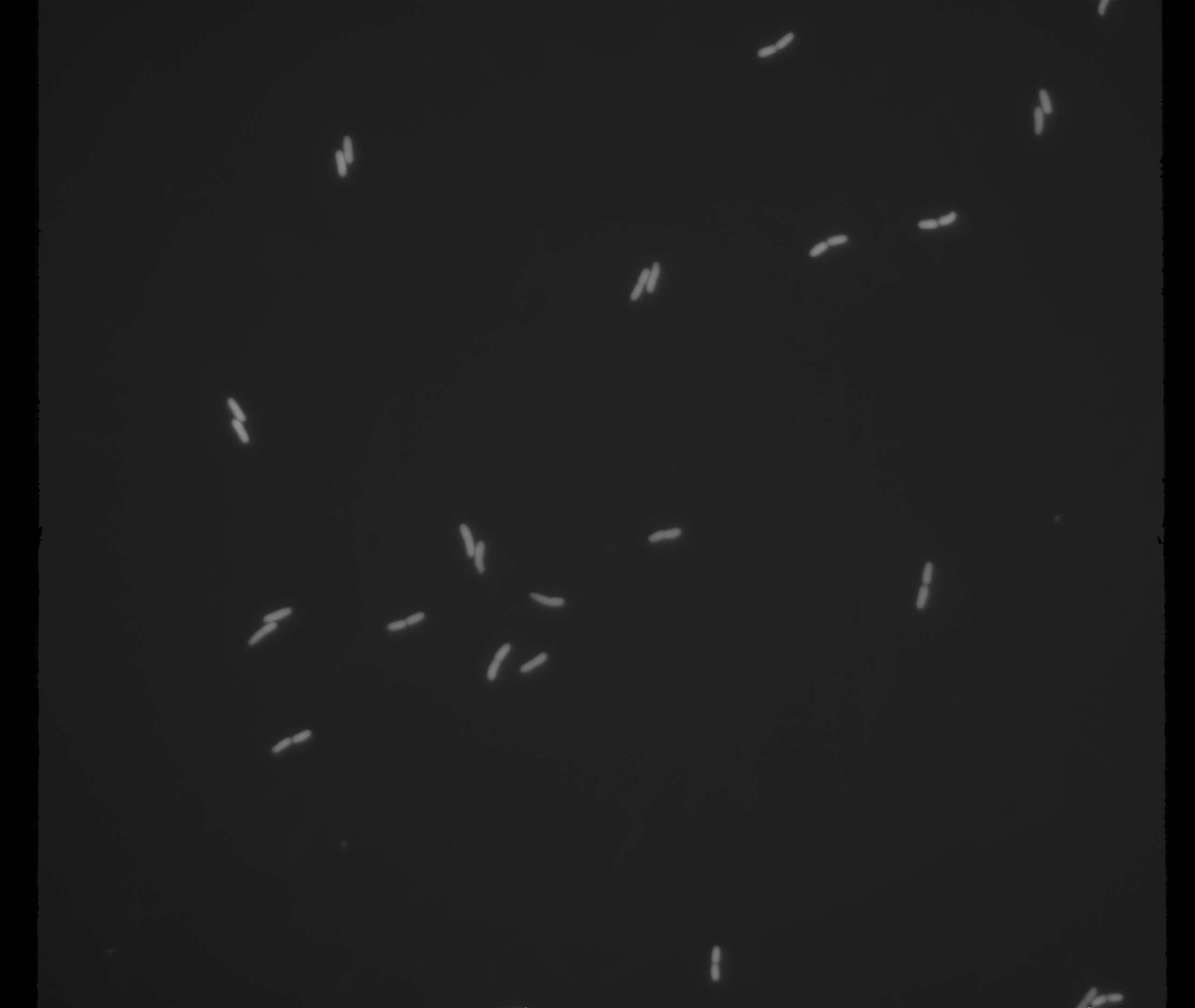

Supplement: Supplementary file 3 — Source data Fig. 2 [file 44318_2025_534_MOESM3_ESM.zip › Figure 2/2A/V316A/img_000000000_EGFP_000.tif]

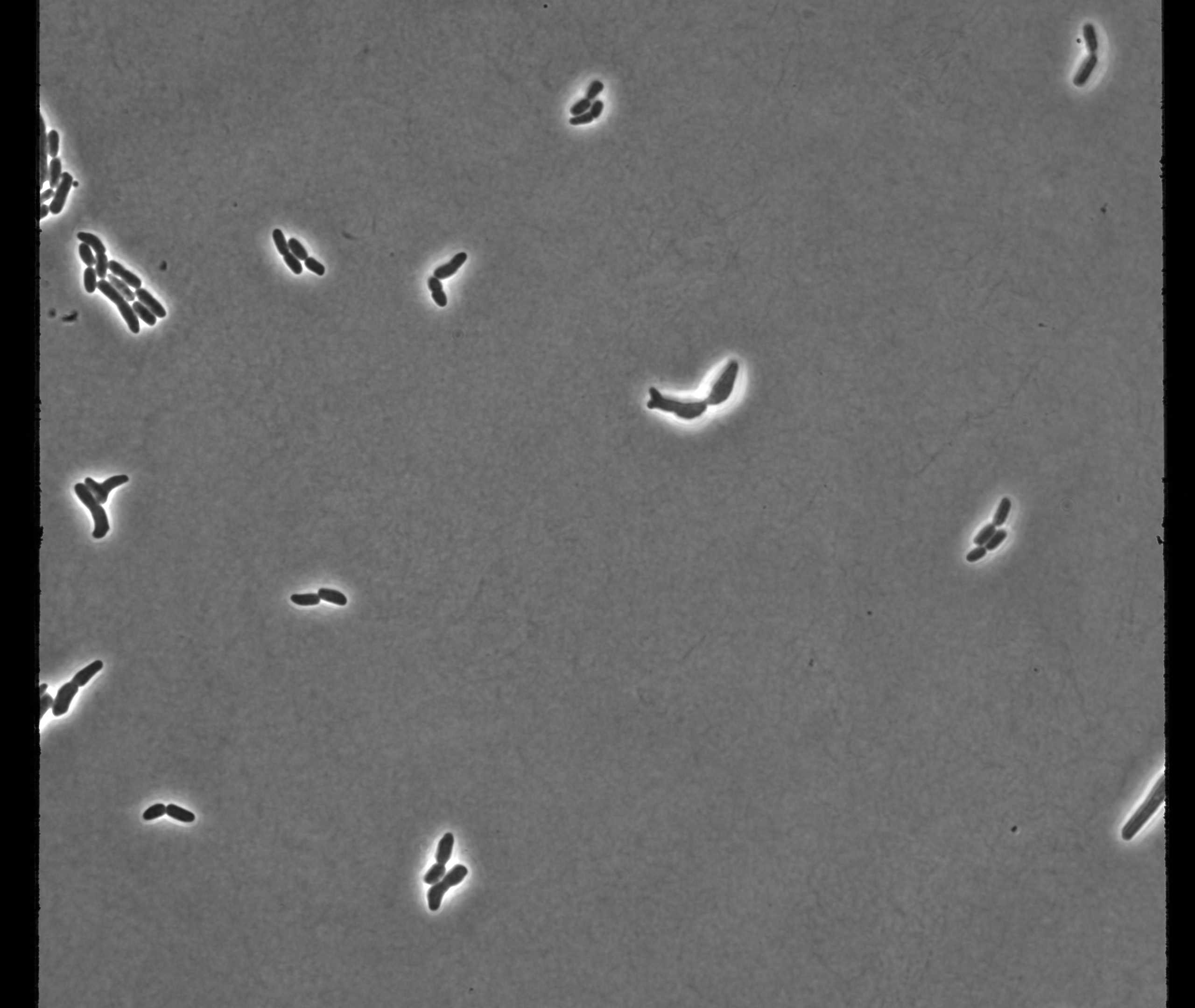

Supplement: Supplementary file 3 — Source data Fig. 2 [file 44318_2025_534_MOESM3_ESM.zip › Figure 2/2A/P314L/img_000000000_Phase_000.tif]

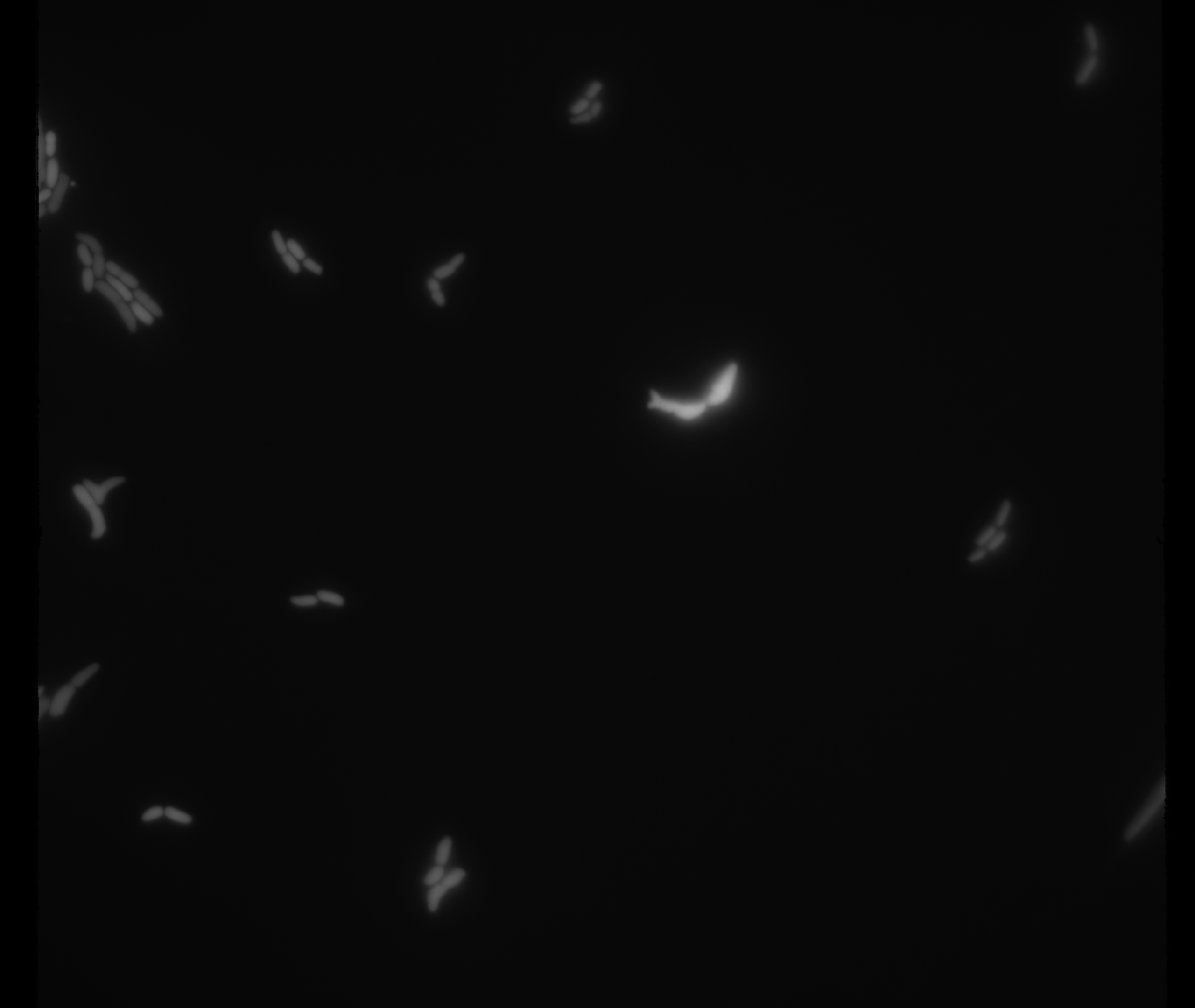

Supplement: Supplementary file 3 — Source data Fig. 2 [file 44318_2025_534_MOESM3_ESM.zip › Figure 2/2A/P314L/img_000000000_EGFP_000.tif]

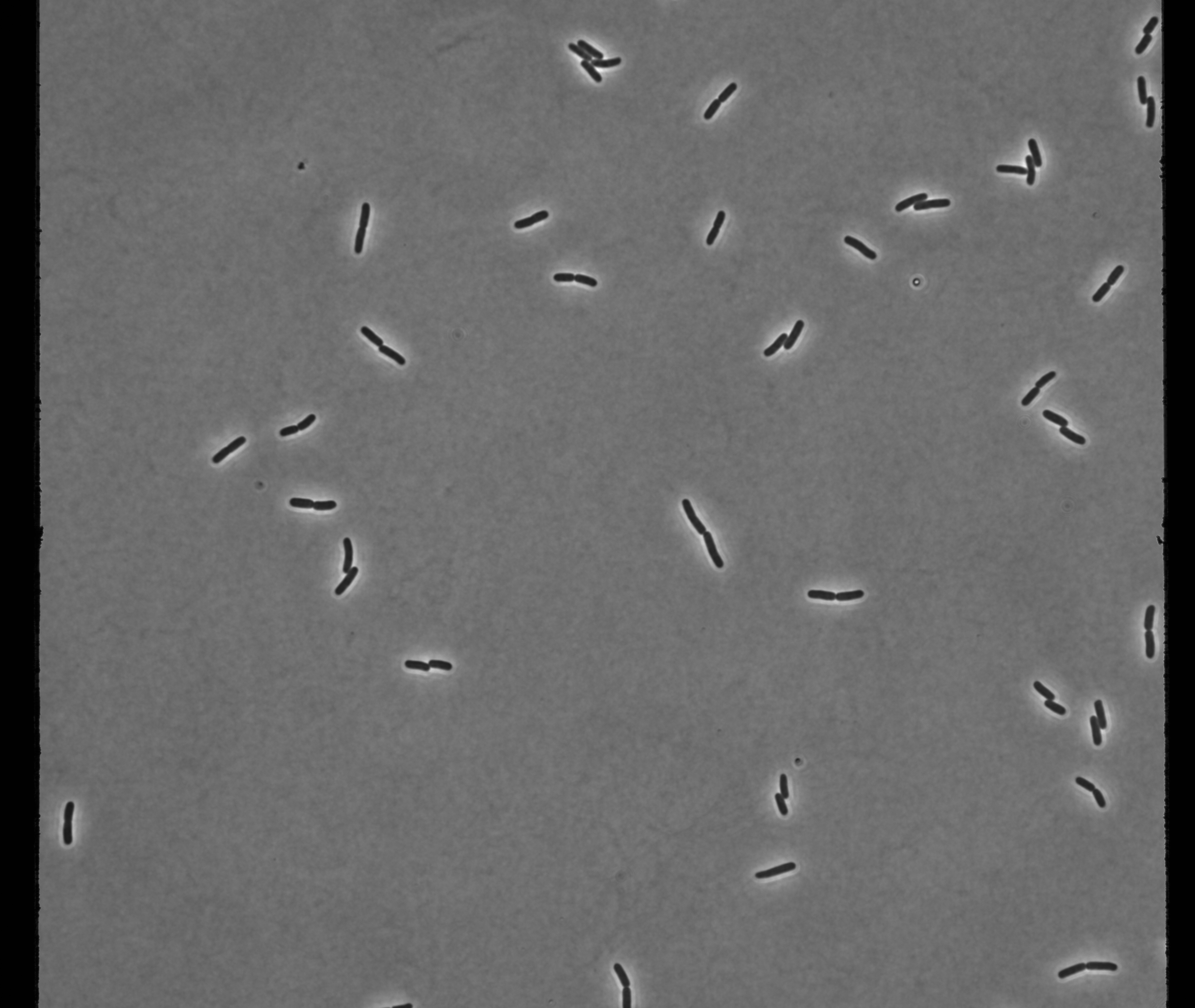

Supplement: Supplementary file 3 — Source data Fig. 2 [file 44318_2025_534_MOESM3_ESM.zip › Figure 2/2A/WT/img_000000000_Phase_000.tif]

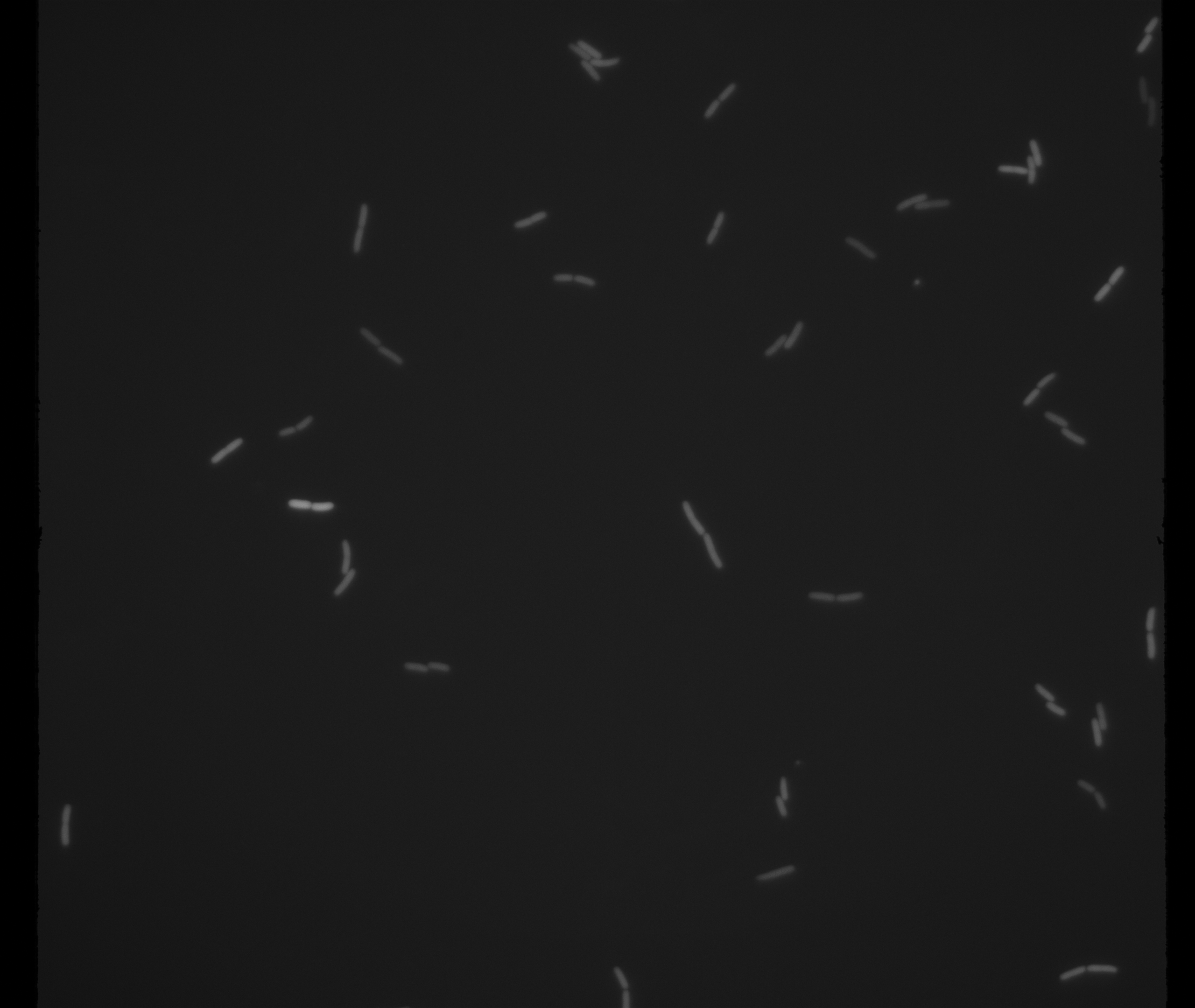

Supplement: Supplementary file 3 — Source data Fig. 2 [file 44318_2025_534_MOESM3_ESM.zip › Figure 2/2A/WT/img_000000000_EGFP_000.tif]

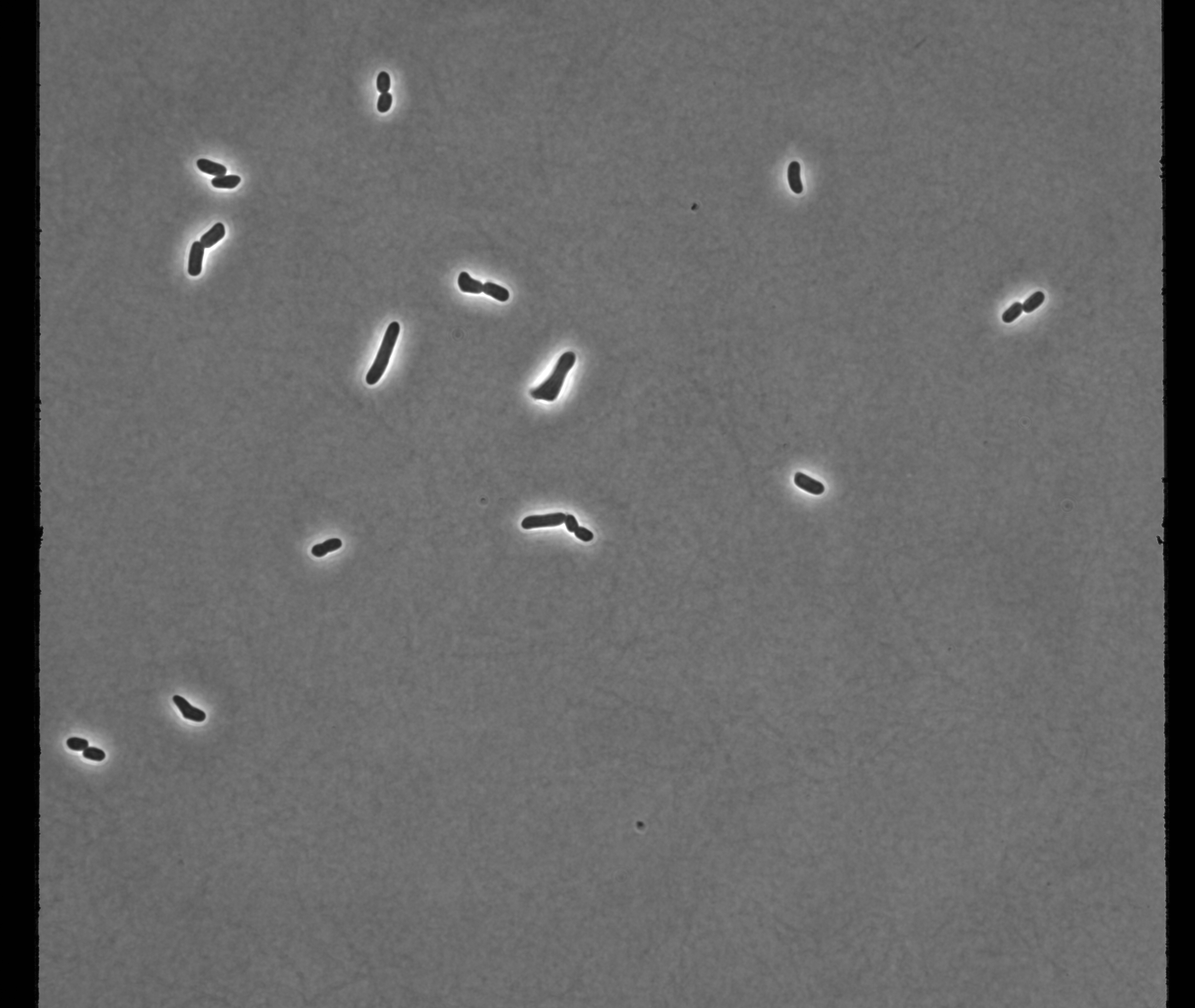

Supplement: Supplementary file 3 — Source data Fig. 2 [file 44318_2025_534_MOESM3_ESM.zip › Figure 2/2A/R193C/img_000000000_Phase_000.tif]

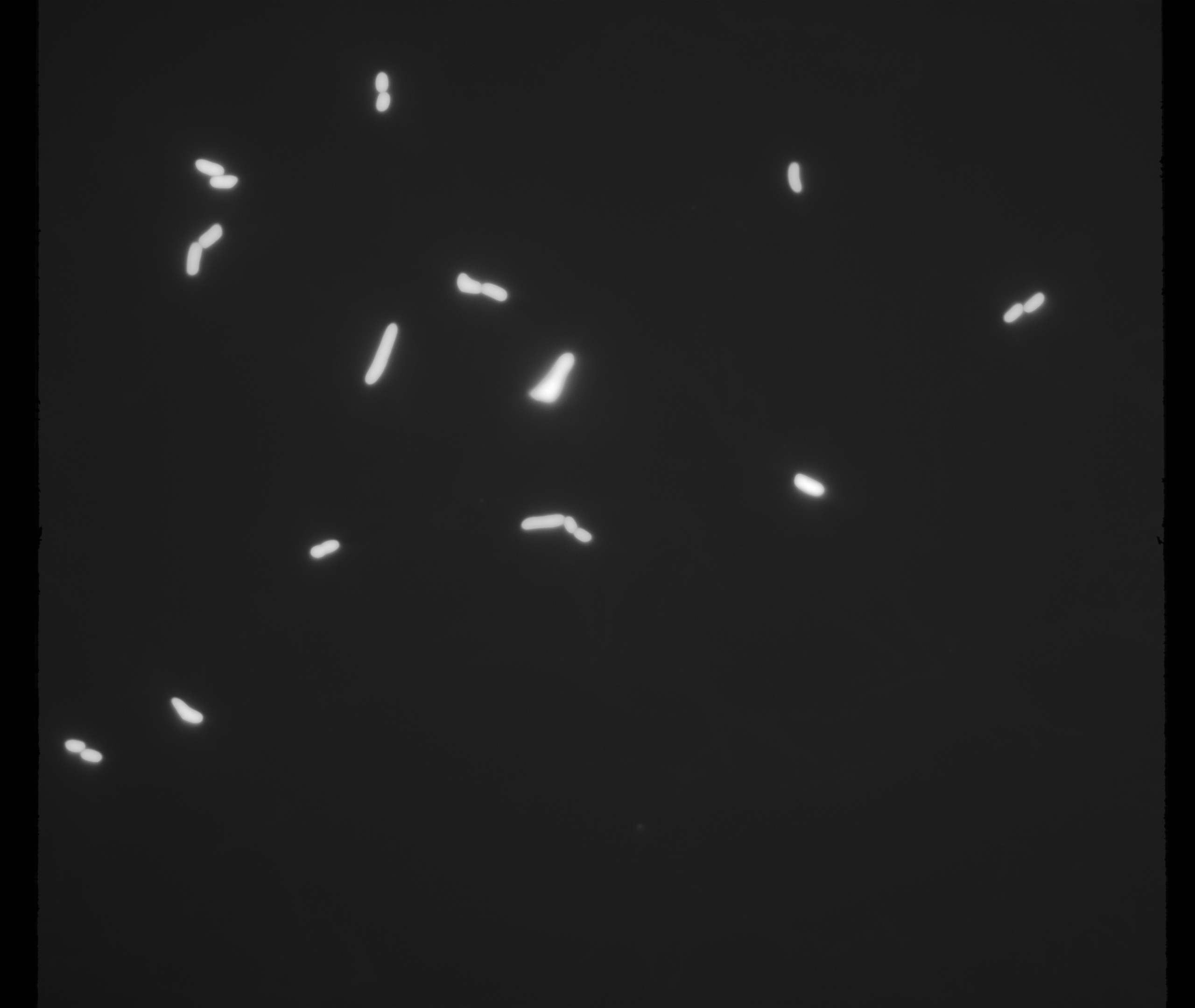

Supplement: Supplementary file 3 — Source data Fig. 2 [file 44318_2025_534_MOESM3_ESM.zip › Figure 2/2A/R193C/img_000000000_EGFP_000.tif]

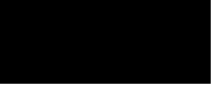

Supplement: Supplementary file 4 — Source data Fig. 3 [file 44318_2025_534_MOESM4_ESM.zip › Figure 3/3B/rcsF-1_fluor.tif]

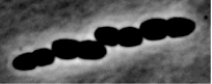

Supplement: Supplementary file 4 — Source data Fig. 3 [file 44318_2025_534_MOESM4_ESM.zip › Figure 3/3B/rcsF-1_phase.tif]

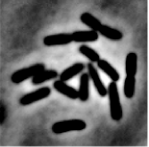

Supplement: Supplementary file 4 — Source data Fig. 3 [file 44318_2025_534_MOESM4_ESM.zip › Figure 3/3B/WT-1_phase.tif]

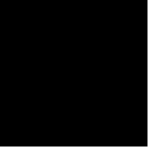

Supplement: Supplementary file 4 — Source data Fig. 3 [file 44318_2025_534_MOESM4_ESM.zip › Figure 3/3B/WT-1_fluor.tif]

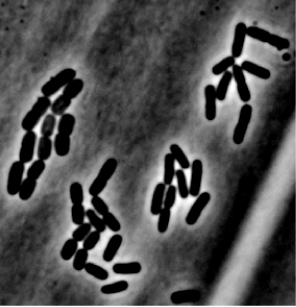

Supplement: Supplementary file 4 — Source data Fig. 3 [file 44318_2025_534_MOESM4_ESM.zip › Figure 3/3B/WT-2_phase.tif]

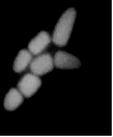

Supplement: Supplementary file 4 — Source data Fig. 3 [file 44318_2025_534_MOESM4_ESM.zip › Figure 3/3B/WT-4_fluor.tif]

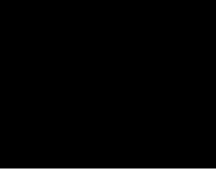

Supplement: Supplementary file 4 — Source data Fig. 3 [file 44318_2025_534_MOESM4_ESM.zip › Figure 3/3B/rcsF-3_fluor.tif]

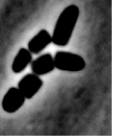

Supplement: Supplementary file 4 — Source data Fig. 3 [file 44318_2025_534_MOESM4_ESM.zip › Figure 3/3B/WT-4_phase.tif]

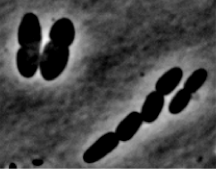

Supplement: Supplementary file 4 — Source data Fig. 3 [file 44318_2025_534_MOESM4_ESM.zip › Figure 3/3B/rcsF-3_phase.tif]

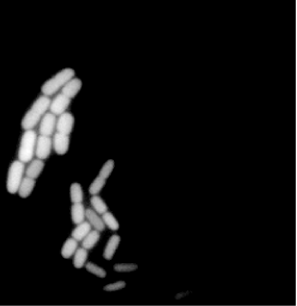

Supplement: Supplementary file 4 — Source data Fig. 3 [file 44318_2025_534_MOESM4_ESM.zip › Figure 3/3B/WT-2_fluor.tif]

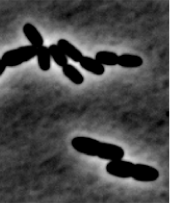

Supplement: Supplementary file 4 — Source data Fig. 3 [file 44318_2025_534_MOESM4_ESM.zip › Figure 3/3B/rcsF-4_phase.tif]

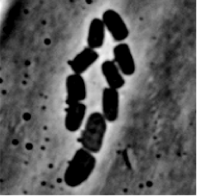

Supplement: Supplementary file 4 — Source data Fig. 3 [file 44318_2025_534_MOESM4_ESM.zip › Figure 3/3B/WT-3_phase.tif]

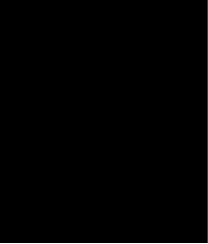

Supplement: Supplementary file 4 — Source data Fig. 3 [file 44318_2025_534_MOESM4_ESM.zip › Figure 3/3B/rcsF-2_fluor.tif]

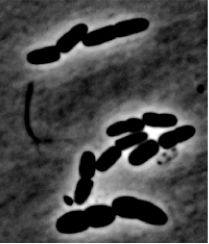

Supplement: Supplementary file 4 — Source data Fig. 3 [file 44318_2025_534_MOESM4_ESM.zip › Figure 3/3B/rcsF-2_phase.tif]

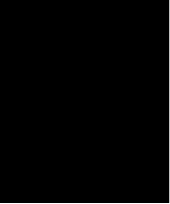

Supplement: Supplementary file 4 — Source data Fig. 3 [file 44318_2025_534_MOESM4_ESM.zip › Figure 3/3B/rcsF-4_fluor.tif]

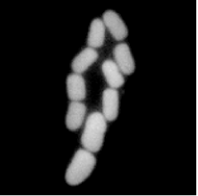

Supplement: Supplementary file 4 — Source data Fig. 3 [file 44318_2025_534_MOESM4_ESM.zip › Figure 3/3B/WT-3_fluor.tif]

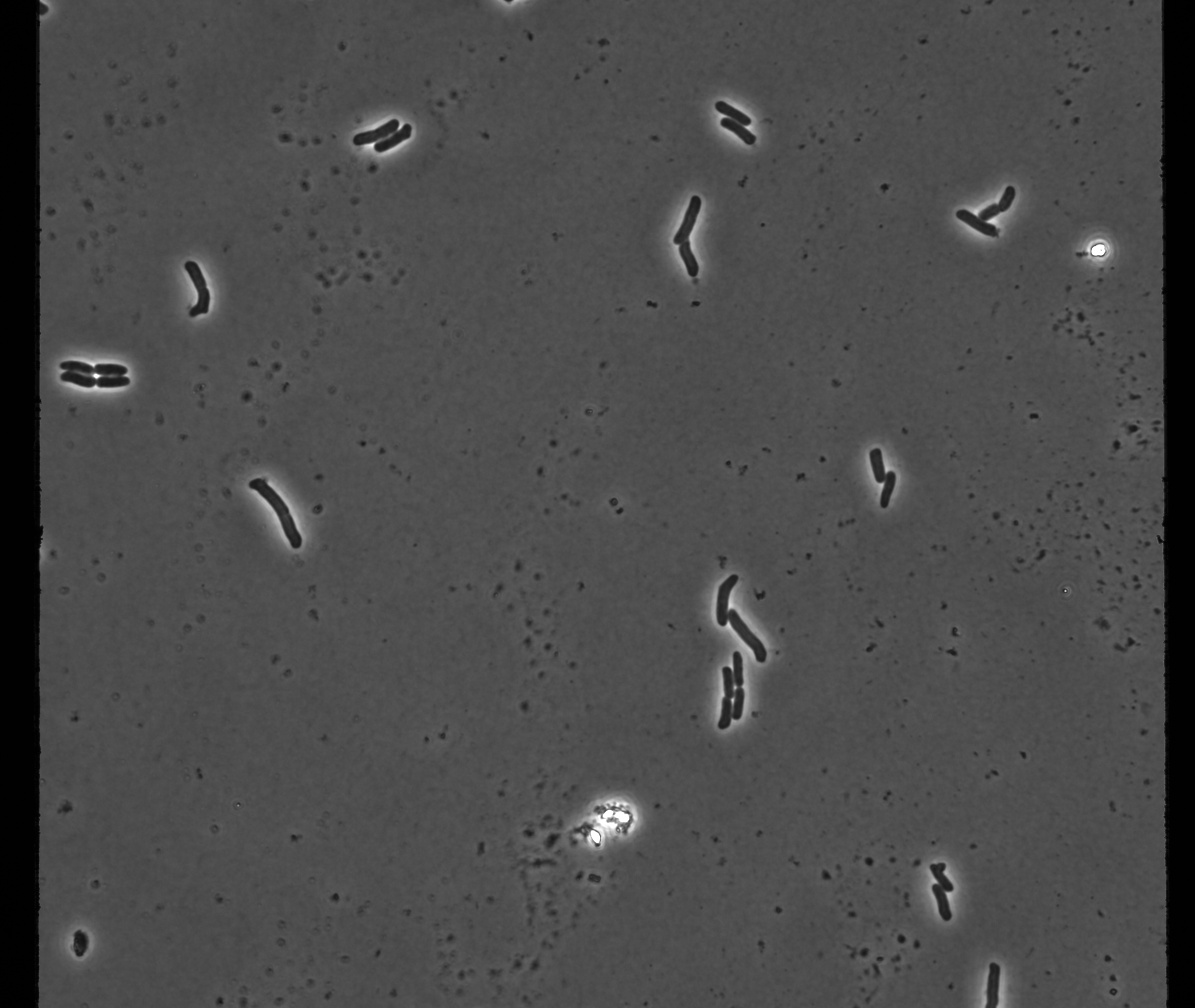

Supplement: Supplementary file 7 — EV Figure Source Data [file 44318_2025_534_MOESM7_ESM.zip › EVFigures/Figure EV2/EV2B/1ugmL.tif]

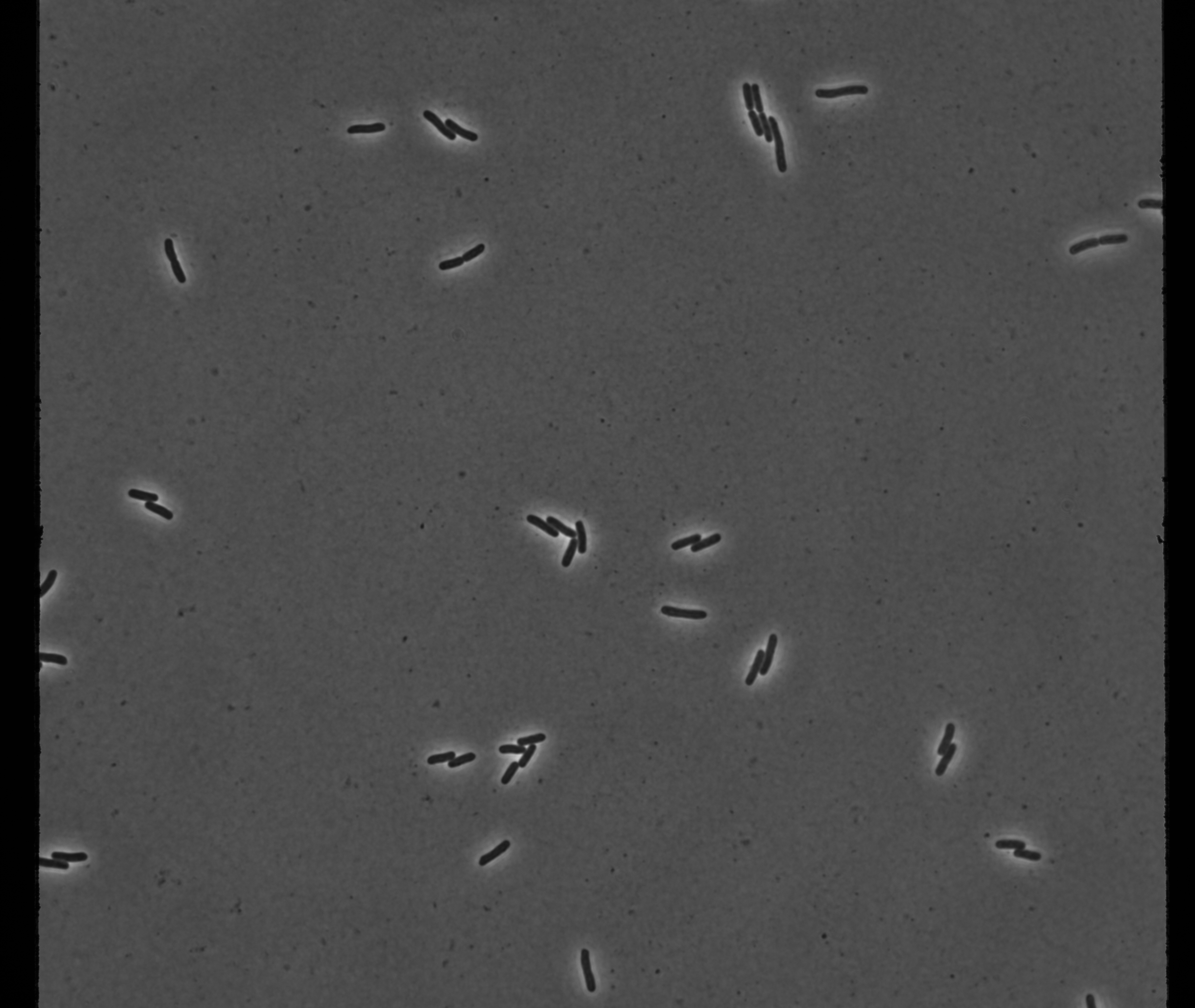

Supplement: Supplementary file 7 — EV Figure Source Data [file 44318_2025_534_MOESM7_ESM.zip › EVFigures/Figure EV2/EV2B/0ugmL.tif]

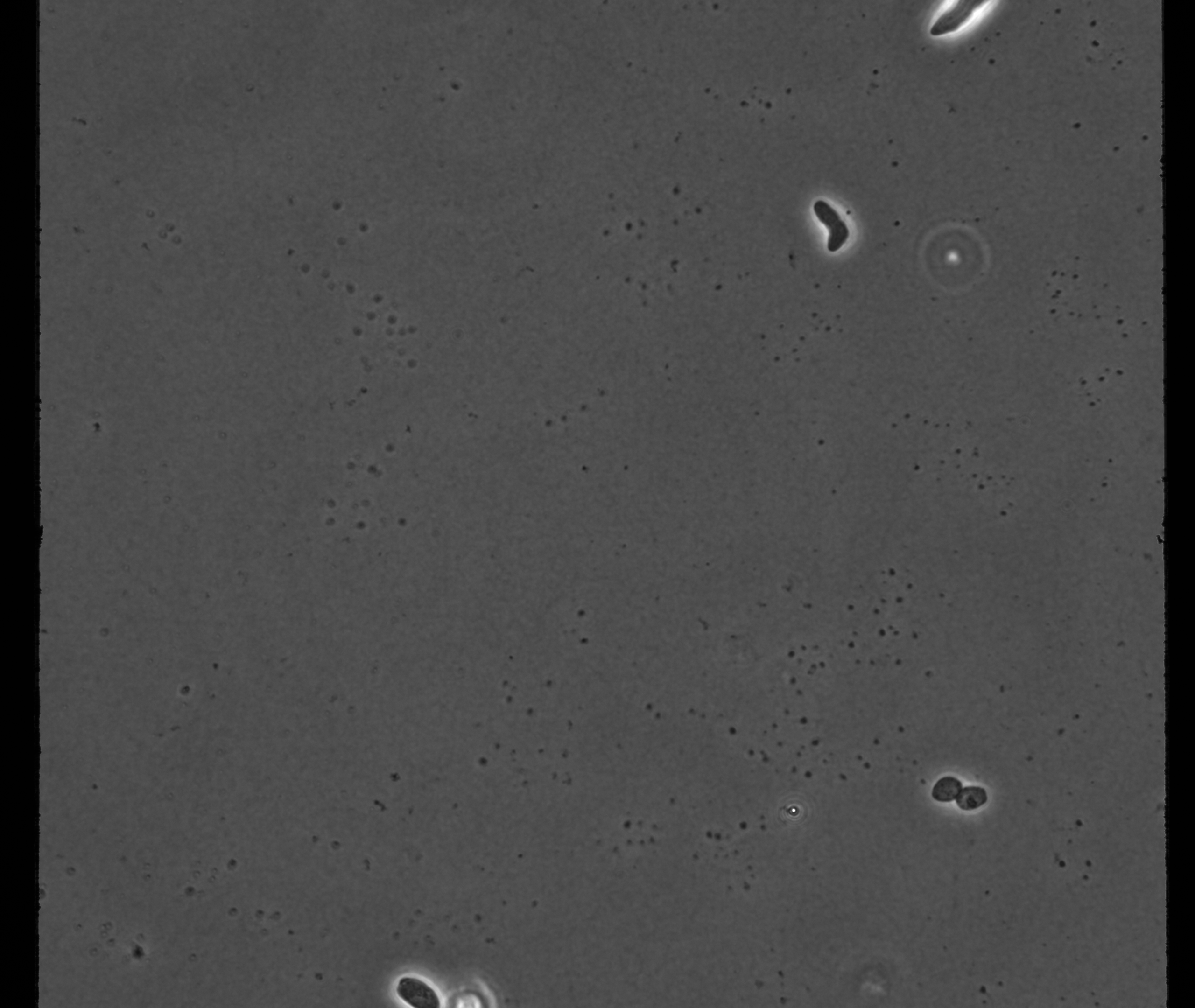

Supplement: Supplementary file 7 — EV Figure Source Data [file 44318_2025_534_MOESM7_ESM.zip › EVFigures/Figure EV2/EV2B/2ugmL.tif]
