# Supplementary material for: Bacterial cell widening alters periplasmic size and activates envelope stress responses
Source: EMBO J. 2025 Sep 3;44(20):5816–33. doi: 10.1038/s44318-025-00534-w (PMC12528386; doi:10.1038/s44318-025-00534-w)
Supplement: Supplementary file 6 — Source data Fig. 5 [file 44318_2025_534_MOESM6_ESM.zip › Figure 5/5B/raw western sucrose gradient RcsF+7.pdf]

RcsF +7

contrasted for RcsF and DsbD

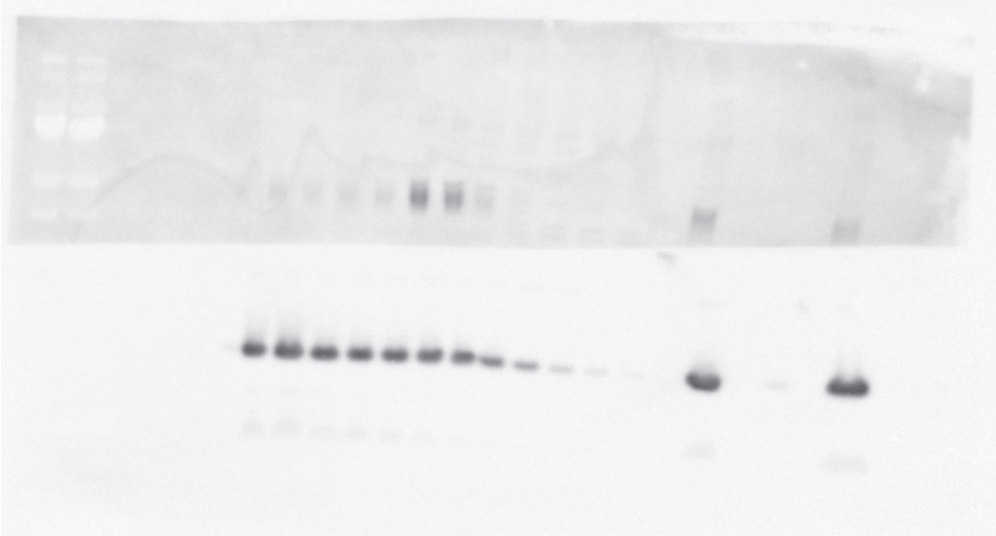

DsbD

RcsF

RcsF +7

contrasted for Lpp

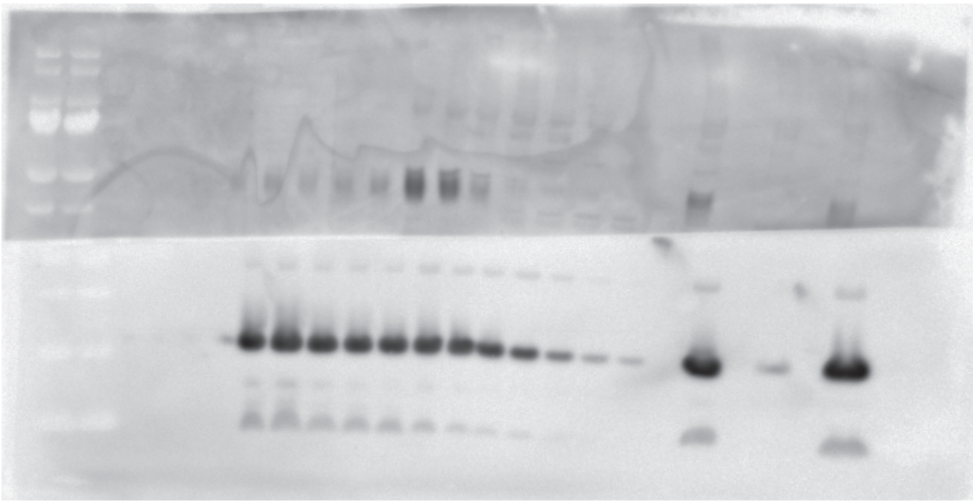

Lpp

RcsF WT

contrasted for RcsF and DsbD

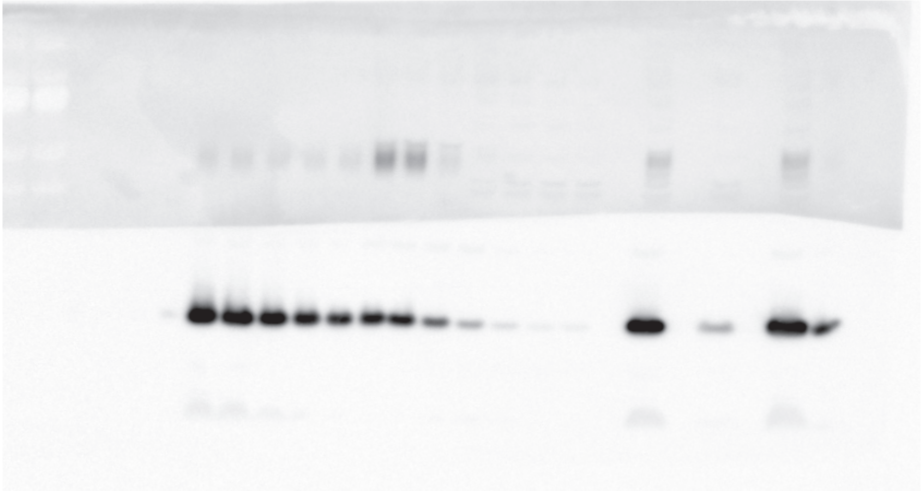

DsbD

RcsF

RcsF WT

CONTRASTED FOR Lpp

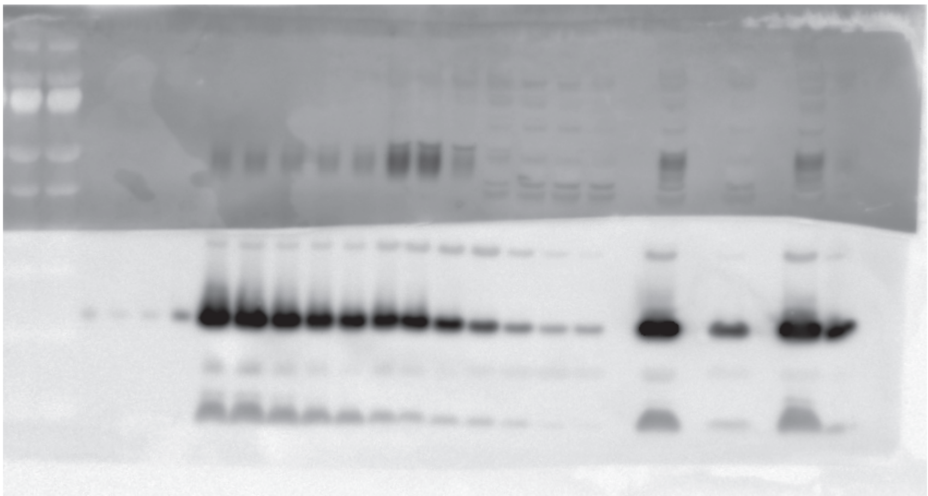

Lpp
