# Supplementary material for: Bacterial cell widening alters periplasmic size and activates envelope stress responses
Source: EMBO J. 2025 Sep 3;44(20):5816–33. doi: 10.1038/s44318-025-00534-w (PMC12528386; doi:10.1038/s44318-025-00534-w)
Supplement: Supplementary file 8 — Expanded View Figures [file 44318_2025_534_MOESM8_ESM.pdf]

# Expanded View Figures

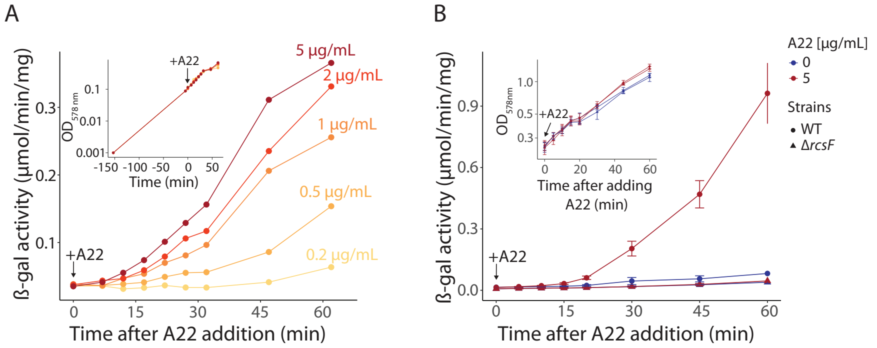


**Figure EV1. Activation of the Rcs system during A22 treatment is dose dependent.**

(**A**) The Rcs system is activated faster at higher A22 concentration. Activation was measured by monitoring induction of chromosomal *rprA::lacZ*. Cells were treated with A22 at $OD_{578\ nm} = 0.3$. Inset: growth was unaffected by A22 addition. (**B**) The Rcs system was activated in wild-type but not Δ*rcsF* cells. Data are $n = 4$ biological replicates of the experiment in Fig. 1A. Data points are mean±1 SD. Source data are available online for this figure.

 © The Author(s)

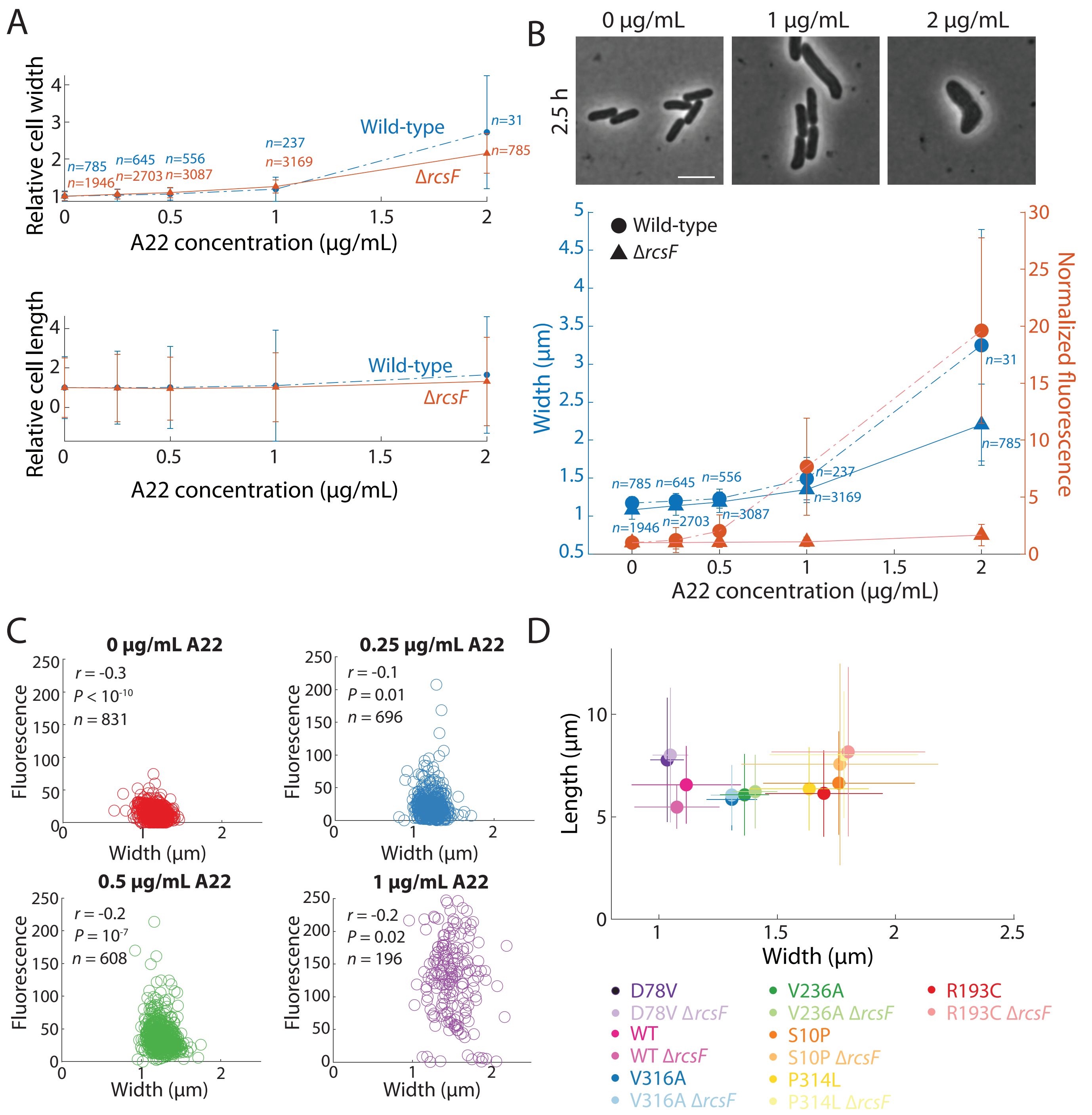


**Figure EV2.** ***rprA*-msfGFP expression is dependent on Rcs activation and is not strongly correlated with natural width variation across a population.**

(**A**) Dimensions of A22-treated wild-type and Δ*rcsF* cells after 2.5 h of A22 treatment, measured relative to the control without treatment. A22-induced changes were not dependent on *rcsF*. Note that *rcsF* cells were more sensitive to A22, and started dying at the A22 concentration 2 μg/mL. For 2 μg/mL A22, wild-type cells increased in width and length more than Δ*rcsF* cells ($P < 0.001$ for both cases, two-tailed Student's *t* tests). The shape of wild-type cells at this concentration was severely deformed from rods and hence had to be excluded from analyses, as length and width could not be defined. Δ*rcsF* cells died before their shape was severely deformed. Data points are mean ± 1 SD, and *n* denotes the number of cells analyzed for each condition. (**B**) Top: images of wild-type cells after 2.5 h of treatment with 0, 1, or 2 μg/mL A22. Scale bar: 5 μm. Bottom: msfGFP intensity from an *rprA* reporter on plasmid pMZ13, as measured via single-cell imaging after 2.5 h of A22 treatment, increased with increasing A22 concentration in wild-type but not Δ*rcsF* cells. For non-zero A22 concentrations, $P = 10^{-10}$ for fluorescence intensity between WT and Δ*rcsF* strains, two-tailed Student's *t* tests. *n* denotes number of cells analyzed for each condition – dying (Δ*rcsF* cells) and especially, severe cell deformation (WT) decreased the number of cells analyzed at the highest A22 concertation. Data points are mean±1 SD. (**C**) The natural variation in cell width across each population after 2.5 h of A22 treatment was not strongly correlated with msfGFP intensity from the *rprA* promoter on pMZ13. *r*: Pearson's correlation coefficient. *P* values are from two-tailed Student's *t* tests. (**D**) No obvious correlations were observed between the cell width and length of MreB mutants. Data points are mean± 1 SD, with $n > 73$ cells for each strain. Source data are available online for this figure.

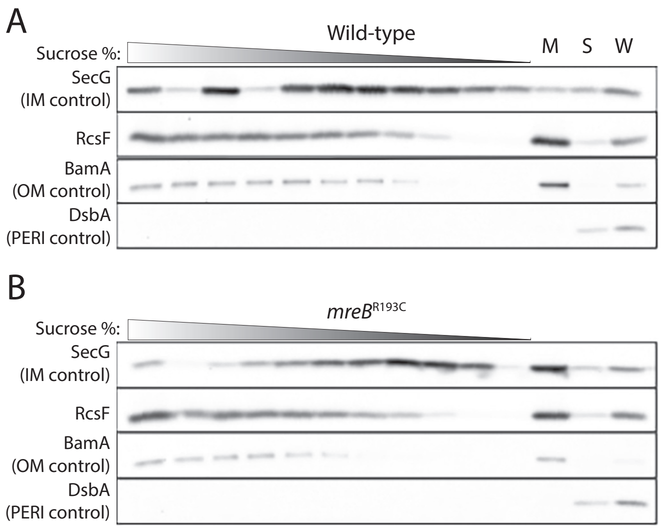


**Figure EV3. RcsF is localized to the outer membrane.**

Sucrose-gradient fractionation shows that RcsF is localized to the outer membrane in both wild-type (**A**) and *mreB*$^{R193C}$ (**B**) cells. Immunoblotting after sucrose-gradient fractionation of RcsF and other control proteins (SecG for the inner membrane, BamA for the outer membrane, and DsbA for the periplasm). OM: outer membrane, IM: inner membrane, PERI: periplasm, M: total membrane sample prior to fractionation, S: soluble non-membrane fraction, W: whole-cell lysate.

 © The Author(s)

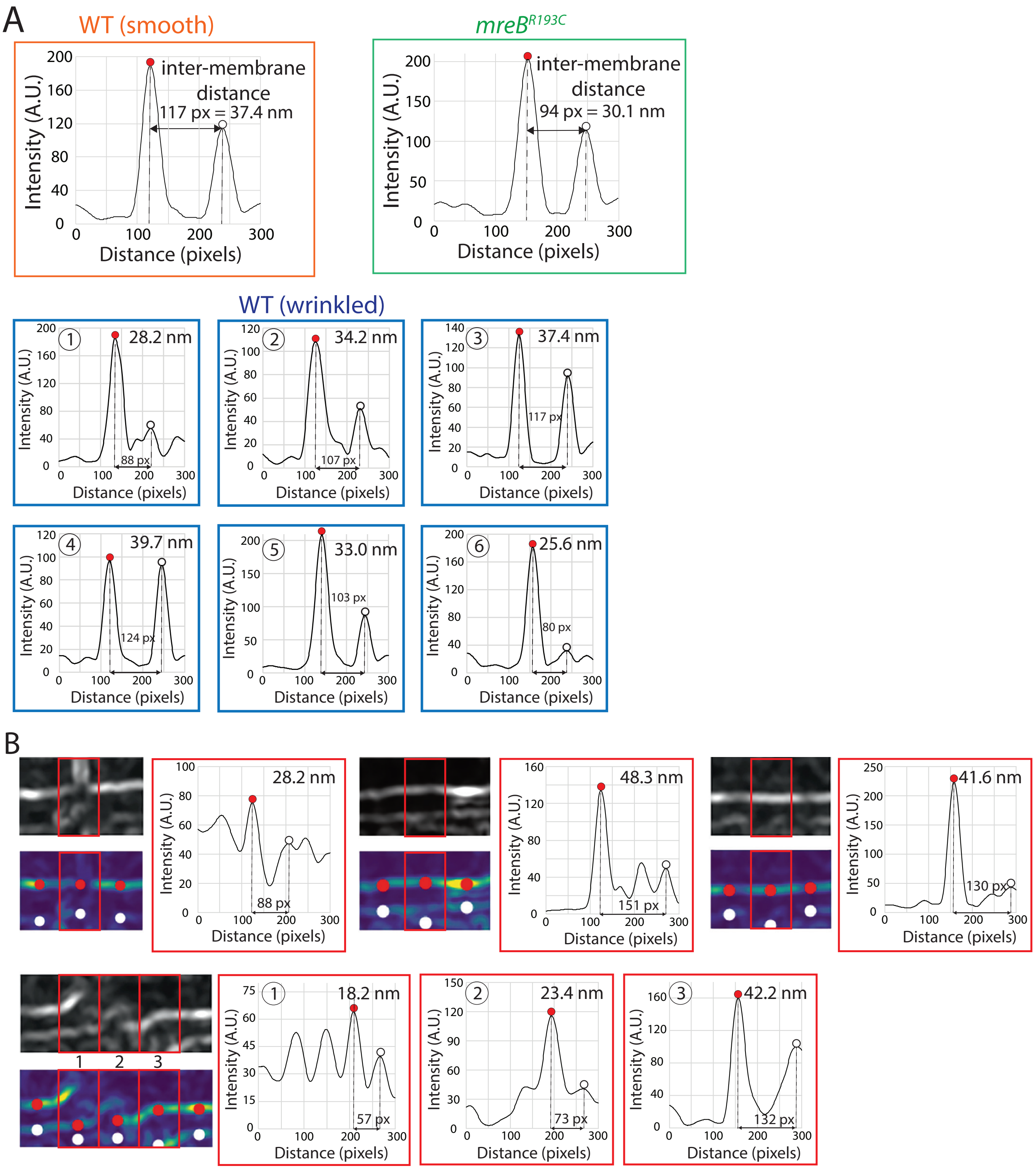


**Figure EV4. Intermembrane distance is calculated based on the distance between bright cross-sections identified as membranes in processed EM images.**

(**A**) Peaks were identified in electron microscopy image intensity averaged across a 50-nm segment in the direction perpendicular to the membranes. The two highest peaks correspond to the middle of the outer membrane (left peak, red dot) and inner membrane (right peak, white dot). Shown are examples corresponding to the boxes in Fig. 4D (wild-type straight membrane, orange box; wild-type wrinkled membrane, cyan boxes 1–6; *mreB*$^{R193C}$ straight membrane, green box). px, pixel. (**B**) Examples of measurements visually identified as erroneous and hence removed due to multiple reasons. Top, from left to right: membrane was discontinuous, a high-contrast object was located in one membrane, insufficient contrast in one membrane. Bottom: incorrect blending of two cryo-EM fields of view. px, pixel.

© The Author(s)	

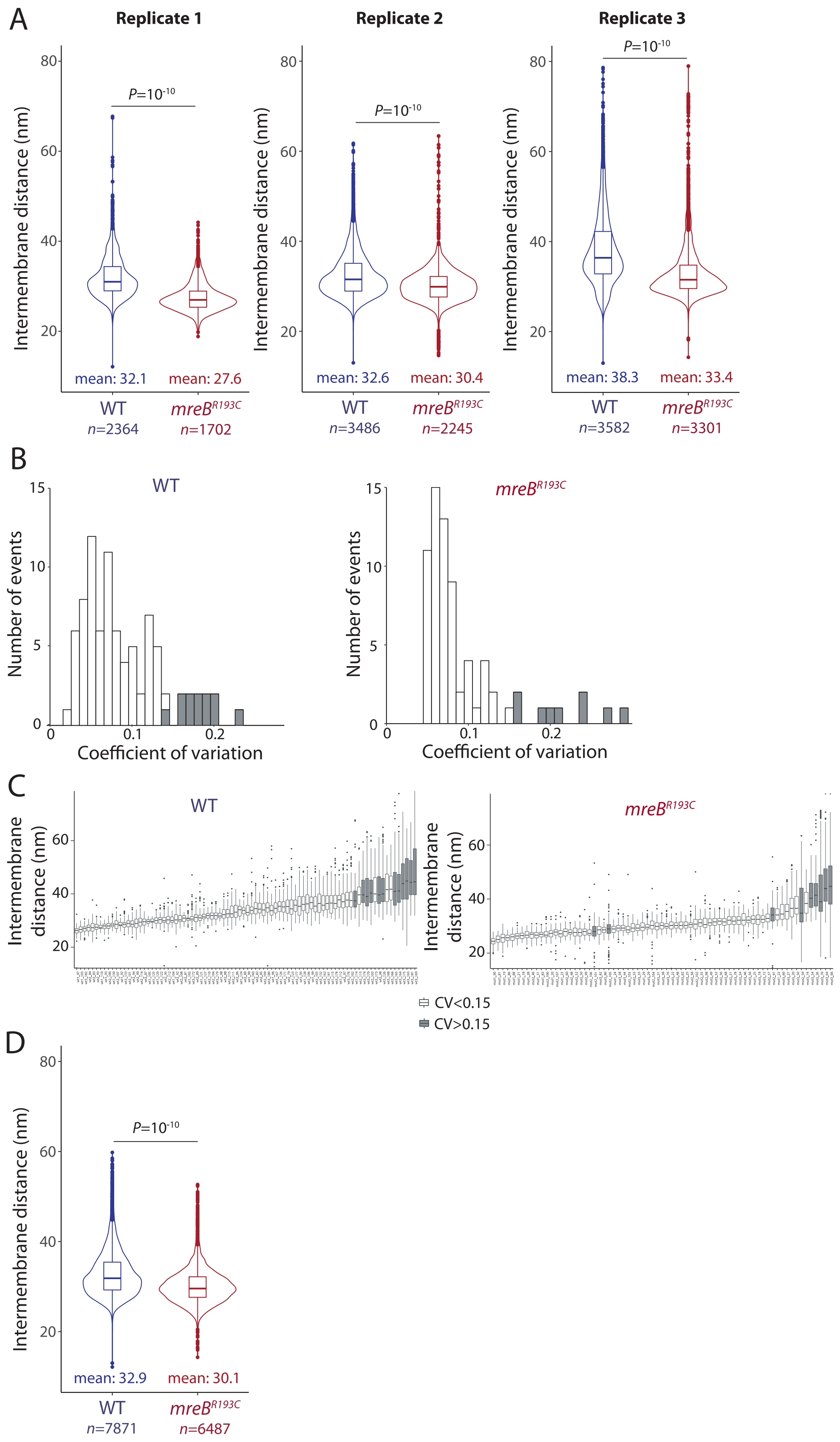
A
Replicate 1
Replicate 2
Replicate 3
Intermembrane distance (nm)
P=10-10
mean: 32.1
mean: 27.6
mean: 32.6
mean: 30.4
mean: 38.3
mean: 33.4
WT
mreBR193C
n=2364
n=1702
n=3486
n=2245
n=3582
n=3301
B
WT
mreBR193C
Number of events
Coefficient of variation
C
Intermembrane distance (nm)
CV<0.15
CV>0.15
D
mean: 32.9
mean: 30.1
n=7871
n=6487

 © The Author(s)

**Figure EV5. The difference in intermembrane distance between wild-type and *mreB*$^{R193C}$ cells is consistent, even though intermembrane distance can vary within a cell, between cells in a population, and between biological replicates.**

(**A**) Intermembrane distance was consistently higher in wild-type cells compared with *mreB*$^{R193C}$ cells across three biological replicates derived from the same experiment as in Fig. 4B. Means are in nm. *n* represents the number of points at which measurements were made, for ~30 cells of each strain in each replicate. *P* values shown are calculated from two-tailed Student's *t* tests. (**B**) The distribution of coefficients of variation (CV) across wild-type (left) and *mreB*$^{R193C}$ (right) cells. Cells with CV > 15% are colored in gray. All three biological replicates were included. (**C**) Intermembrane distances within each wild-type (left) and *mreB*$^{R193C}$ (right) cell, sorted by mean intermembrane distance. Cells with CV > 15% are colored in gray. (**D**) Intermembrane distance was consistently higher in wild-type cells compared with *mreB*$^{R193C}$ cells when considering only cells with CV < 15% in the dataset presented in Fig. 4B. *n* represents the number of points at which measurements were made, for 74 wild-type and 62 *mreB*$^{R193C}$ cells from three biological replicates pooled together, the *P* value shown was calculated from two-tailed Student's *t* test. Violin plots in (**A**, **D**) show the smoothened density of measurements. Box plots in (**A**, **C**, **D**) show the median and first and third quartiles, and the whiskers are 1.5 interquartile ranges from the corresponding quartiles. Data points outside the whiskers are plotted as individual points. Source data are available online for this figure.

© The Author(s) 

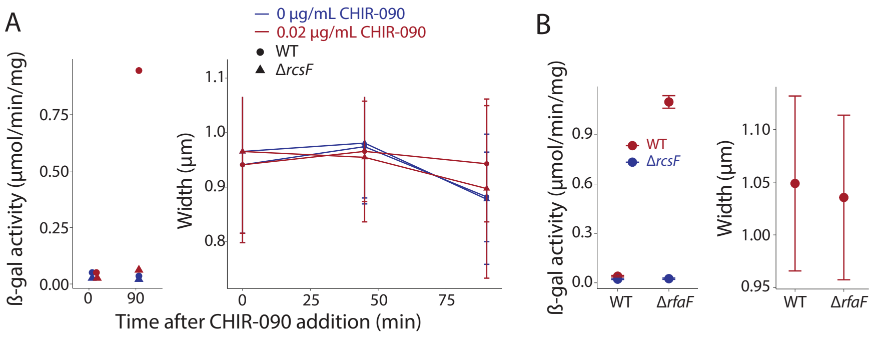


**Figure EV6. Cell width is not affected by CHIR-090 treatment or deletion of *rfaF*.**

(**A**) Treatment with 0.02 μg/mL CHIR-090 activates the Rcs pathway (left, $n = 1$ replicate) even though cell width is unaffected (right, $n > 200$ cells for each data point). (**B**) Left: the Rcs pathway is activated in Δ*rfaF* cells. Δ*rfaF* cells had significantly higher Rcs pathway activation compared to wild-type (WT) or Δ*rcsF* cells ($P = 10^{-5}$, $10^{-6}$, and $10^{-6}$, compared to WT, Δ*rcsF*, and Δ*rfaF* Δ*rcsF*, $n = 3$ replicates, two-tailed Student's *t* tests). Right: cell width is largely unaffected by *rfaF* deletion ($n = 1142$ or 926 cells for WT and Δ*rfaF*, respectively). For width measurements in (**A**, **B**), data are mean values and error bars represent 1 SD. Source data are available online for this figure.

 © The Author(s)
